# Short-term glycemic variability and the risk of adverse ICU outcomes in critically ill patients: a systematic review and meta-analysis

**DOI:** 10.3389/fnut.2026.1808757

**Published:** 2026-06-09

**Authors:** Zhongyi Wu, Jianzheng Cai, Mingjing Fang, Weixia Yu, Cen Bi, Fang Shi, Xiaoyan Lu

**Affiliations:** 1 Department of Critical Care Medicine, The First Affiliated Hospital of Soochow University, Suzhou, China; 2 Department of Nursing, The First Affiliated Hospital of Soochow University, Suzhou, China; 3 Department of Nursing, Suzhou Hospital Affiliated to Nanjing Medical University, Suzhou, China

**Keywords:** critical illness, glycemic variability, intensive care unit, prognosis, systematic review and meta-analysis

## Abstract

**Objective:**

To quantify associations between short-term glycemic variability (GV) metrics and multidimensional adverse outcomes in critically ill patients.

**Methods:**

We searched PubMed, EMBASE, and Web of Science from inception to August 16, 2025. We included observational studies of adult ICU patients reporting associations between short-term GV and adverse outcomes. Random-effects models were used for all meta-analyses. Where feasible, effect estimates were standardized to a relative risk (RR) comparing the highest versus lowest quartiles of GV.

**Results:**

We included 36 studies (123,911 patients), and 25 were meta-analyzed. ICU mortality was associated with standard deviation (SD; RR = 2.29, 95% CI 1.71–3.07) and mean absolute glucose change (MAG; RR = 2.24, 95% CI 1.19–4.23). Hospital mortality was associated with coefficient of variation (CV, RR = 1.39, 95% CI 1.05–1.85) and SD (RR = 2.26, 95% CI 1.19–4.30). 28/30-day mortality was associated with CV (RR = 1.34, 95% CI 1.10–1.63) and mean amplitude of glycemic excursions (MAGE; RR = 2.05, 95% CI 1.52–2.77), and MAGE also predicted 90-day mortality (RR = 2.90, 95% CI 1.96–4.30). Furthermore, each unit increase in SD predicted higher infection risk (OR = 1.02, 95% CI 1.01–1.04) but not neurological adverse events (OR = 1.23, 95% CI 0.91–1.66).

**Conclusion:**

Short-term GV is a robust predictor of mortality across different follow-up windows and clinical settings, as well as infection-related outcomes. The current findings support using short-term GV as a key prognostic marker to complement mean glucose in intensive care.

**Systematic review registration:**

https://www.crd.york.ac.uk/prospero/display_record.php?ID=CRD420251114266, identifier PROSPERO (CRD420251114266).

## 1 Introduction

Glucose homeostasis is a tightly regulated physiological process, yet it is frequently disrupted during critical illness. In the intensive care unit (ICU), this disruption typically manifests as insulin resistance, stress hyperglycemia (SH), and a subsequent dependence on exogenous insulin for glycemic control (1, 2). Traditionally, ICU glucose management has focused on maintaining mean blood glucose levels within a defined range to mitigate SH-associated infection risks and inflammatory responses while protecting against hypoglycemia-induced neurological injury (3, 4). However, evidence suggests that targeting mean glucose alone is insufficient, as it fails to capture the dynamic nature of glycemic fluctuations and does not fully explain the significant heterogeneity observed in patient outcomes (5).

Glycemic variability (GV), which refers to fluctuations in blood glucose levels over time, has emerged as a critical consideration in glucose management (6, 7). Specifically, long-term GV is characterized by variations in glycated hemoglobin (HbA1c) or fasting glucose over months or years (8). Short-term GV is derived from repeated glucose measurements over a relatively short observation window. It does not have a fixed temporal definition, and is typically assessed using 24-h or multi-day data, reflecting intra-day and inter-day fluctuations (9). In the acute ICU setting, patients experience rapid, frequent shifts in metabolic status that require immediate clinical oversight (10), and short-term GV is increasingly recognized as an important independent predictor of clinical outcomes (11). Mechanistically, acute fluctuations are thought to be more damaging than sustained hyperglycemia (12), as they impair immune function and organ performance by inducing oxidative stress, activating inflammatory pathways, and causing endothelial dysfunction, thereby increasing the clinical burden of critical illness (13, 14).

Multiple systematic reviews have linked elevated short-term GV to increased mortality in specialized populations, such as those with sepsis or acute stroke (15, 16), and a recent systematic review extended these associations to broader critically ill populations (17). However, the current body of evidence remains limited by several methodological gaps. First, existing reviews have focused heavily on standard deviation (SD) and coefficient of variation (CV) as GV metrics, which leaves other validated metrics underexplored. Similarly, research has prioritized mortality, leaving non-mortality endpoints such as infection and neurological complications under-analyzed. Even within the literature on mortality, there is a lack of granular stratification; disparate endpoints (ICU, in-hospital, and 28/30/90-day mortality) tend to be pooled together, which obscures temporal trends and inflates heterogeneity. Finally, previous studies have often failed to apply consistent standardization across different exposure comparison approaches (e.g., per-unit vs. quantile-based comparisons), which compromises the comparability and clinical interpretability of pooled results.

To address these limitations, we conducted a systematic review and meta-analysis to comprehensively evaluate the associations between diverse short-term GV metrics and multidimensional clinical outcomes. Our goal was to provide a more refined, standardized quantitative synthesis that distinguishes between specific mortality windows and extends the analysis to non-survival ICU outcomes, thereby offering a stronger evidence base for risk stratification in the ICU.

## 2 Methods

This review is registered in the International Prospective Register of Systematic Reviews (PROSPERO) (CRD420251114266) and adheres to the Preferred Reporting Items for Systematic Review and Meta-Analyses (PRISMA) and Meta-analysis of Observational Studies in Epidemiology (MOOSE) guidelines (18, 19). Supplementary Table 1 provides the PRISMA checklist.

### 2.1 Search strategy

The search strategy targeted the relationship between GV and adverse ICU outcomes. We searched PubMed, EMBASE, and Web of Science from inception to August 16, 2025. The search terms included “glycemic variability,” “glucose fluctuation,” “critical illness,” “intensive care unit”, and “outcomes”, using both controlled vocabulary and free-text terms. The full search strategy is provided in Supplementary Table 2.

### 2.2 Selection criteria

Eligible studies met the following P–I–O–S criteria:

Population (P): Adult patients (≥18 years) admitted to and managed within the ICU.

Exposure (I): The exposure of interest was short-term GV, whose metrics included SD, CV, mean absolute glucose (MAG), mean amplitude of glycemic excursions (MAGE), and other validated indices quantifying glucose fluctuations (Supplementary Table 3). We excluded studies that assessed variability solely using simple range-based measures (e.g., maximum–minimum difference), as these are sensitive to extreme values and measurement frequency, limiting comparability.

Outcomes (O): Outcomes included ICU-related clinical endpoints such as mortality, infectious complications, organ dysfunction or failure, resource utilization, and other relevant adverse events. Eligible studies must also provide effect estimates suitable for quantitative synthesis. For binary outcomes, they must report odds ratios (OR), relative risks (RR), or hazard ratios (HR) with 95% confidence intervals (CI); for continuous outcomes, *β* coefficients from linear regression models were required.

Study design (S): Only observational studies published in English were included; reviews, commentaries, case reports, conference abstracts, methodological papers, and studies lacking sufficient extractable data were excluded. To avoid duplicate populations, when multiple publications originated from the same dataset, we included the study with the longest follow-up, largest sample size, or most comprehensive outcome reporting.

**Figure 1:**
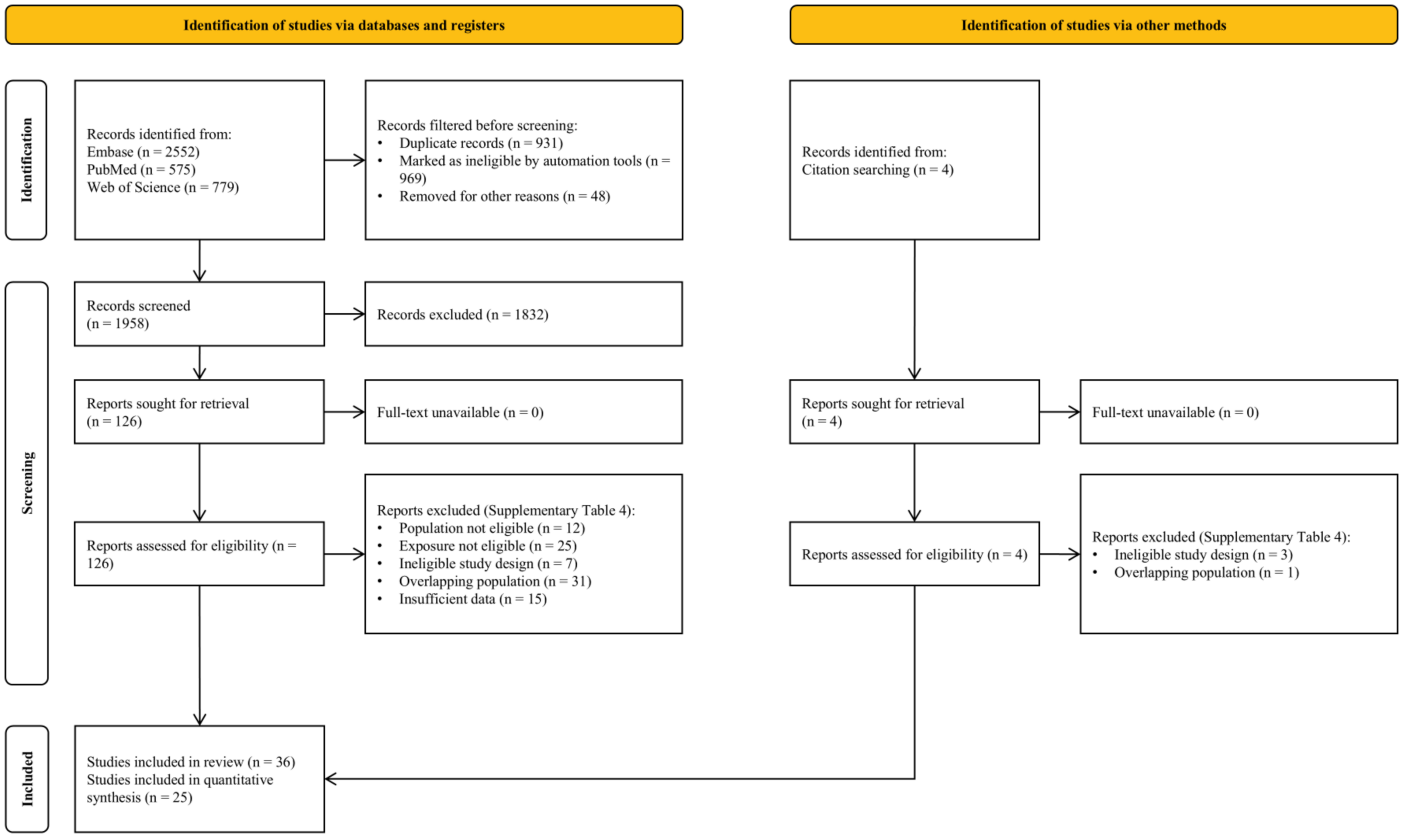
PRISMA flowchart diagram for identification and selection of studies.

To ensure completeness, the primary studies in the citations of the retrieved systematic reviews and meta-analyses were also assessed (Figure 1).

### 2.3 Study selection

Duplicates were removed using EndNote X9. Two reviewers independently screened titles and abstracts, and for the items passing the initial screen, full text was retrieved and assessed. Discrepancies were resolved through discussion or consultation with a third reviewer.

### 2.4 Study quality assessment and risk of bias

Two reviewers independently assessed risk of bias using the Newcastle–Ottawa Scale (NOS) (20). Studies scoring ≥7 (out of 9) were classified as having good methodological quality.

### 2.5 Data extraction

Two investigators independently extracted data using a predefined form; discrepancies were resolved through discussion with a third investigator and by consulting the original reports. To improve comparability across studies and minimize bias arising from different exposure categorizations, we standardized various types of effect measures (HR, RR, OR) and comparison formats (e.g., per-unit increase, per-SD increase, extreme dichotomies, tertiles, quartiles, or quintiles) into a unified RR metric. The estimates were expressed as a comparison between the highest and lowest quartiles (Q4 vs. Q1) (8, 21), reflecting the relative risk difference between high and low exposure levels. This process consisted of two steps.

For exposure contrast harmonization, under the assumption that log-risk estimates follow an approximately normal distribution, the comparison between the highest (Q4) and lowest (Q1) quartiles corresponds to 2.54 times the log-RR per 1-SD increase. Accordingly, scaling factors of 2.54/1.59, 2.54/2.18, and 2.54/2.80 were applied for extreme dichotomies, tertiles, and quintiles, respectively (22).

For effect measure harmonization, HR were treated as approximations of RR (23), and OR were converted to RR using the formula RR = OR/[(1 − *P*₀) + (*P*₀ × OR)], where *P*₀ denotes the outcome incidence in the low-exposure group (24); when *P*₀ was not reported, the overall incidence was used as a substitute.

This standardization enabled quantitative pooling of effect estimates from different GV metrics and comparison schemes on a common scale. When transformation to a unified RR was not feasible, the originally reported estimates were synthesized, provided that GV definitions and outcome measures were comparable. Further details are provided in the Supplementary Methods.

### 2.6 Statistical analysis

We performed a meta-analysis to quantitatively synthesize results when at least two studies evaluated the same GV metric and outcome, with effect estimates pooled separately based on adjustment status. A random-effects model was used to account for heterogeneity in population characteristics, GV definitions, comparison schemes, and outcomes. Pooled effects were expressed as RR with 95% CIs. We conducted subgroup analyses to explore heterogeneity sources where data permitted. Subgroups were defined by relevant variables, including population characteristics, GV measurement window, and outcome categories. To further validate the robustness of the findings, supplementary validation was performed using the originally reported data. Direct pooling of effect measures and original exposure formats was used where applicable; otherwise, only the exposure format was harmonized, with the original effect estimates preserved.

Additionally, we conducted sensitivity analyses to assess robustness where appropriate; for outcomes with 10 or more studies, we evaluated publication bias using funnel plots and Egger’s regression test. For each outcome, the certainty of evidence was appraised using the GRADE approach (25). Evidence strength was rated as high, moderate, low, or very low based on risk of bias, inconsistency, indirectness, imprecision, and publication bias.

## 3 Results

### 3.1 Study selection and characteristics

Database searches yielded 3,910 records. After thorough screening (Figure 1), 36 studies involving 123,911 participants were included (Table 1; the details of exclusions are described in Supplementary Table 4). The included studies were published between 2006 and 2025 and represented diverse geographic regions in Asia (*n* = 18), Europe (*n* = 8), the Americas (*n* = 8), and Oceania (*n* = 3). One study was carried out jointly between Australia and Sweden. Most adopted a retrospective design (*n* = 31) and some were prospective (*n* = 5). Populations were recruited predominantly from general ICUs, with additional data from neurological, emergency, cardiac, trauma, and burn units. Sample sizes ranged from 28 to over 52,000 participants. Most studies reported a mean/median age between 50 and 75 years. The methodological quality of these studies was generally moderate to high, and 35 of the 36 studies achieved an NOS score of 7–9 (Supplementary Table 5).

**Table 1:** Characteristics of the included studies (*n* = 36).

Reference	Region	Study period	Study design^†^	ICU type^†^	Number and characteristics of patients^§^	GV metrics^¶^	Outcome measures^⸸^	Effect estimates^⸸^	Comparison
Ammar et al. (2022) (54)	USA	2013–2018	R	X	5,287	TIR	MR (Hospital)	OR	High vs. low
Bansal et al. (2016) (41)	India	2014	P	C	870	SD	LOS (ICU and hospital) Infection; Readmission; AKI	OR	Per unit
Cai et al. (2020) (27)	China	2014–2016	R	N	158 (M/F 100/58)	SD; CV; MAGE	MR (3 months)	OR	Per unit
Chao et al. (2020) (28)	Taiwan	2014–2015	R	M	452 (M/F 346/106) 71.4 ± 14.7 years	CV; MAGE	MR (30 days)	HR	High vs. low
Dahagam et al. (2011) (29)	USA	2006–2009	R	B	462	CV	ICU-free days; Hospital-free days; Ventilator-free days	β	Per unit
Donati et al. (2014) (50)	Italy	2004–2010	R	X	2,782 (M/F 1886/896) 63 [44, 74] years	GLI	MR (ICU); Infection (ICU-acquired)	OR	Extreme quartiles
Doola et al. (2018) (30)	Australia	2014–2016	R	X	759 (M/F 499/260) 56.9 [43.4, 68.2] years	CV	MR (ICU)	OR	Per unit
Egi et al. (2006) (40)	Australia	2000–2004	R	X	7,049 (M/F 4287/2762) 61 ± 18 years	SD	MR (ICU); MR (Hospital)	OR	Per unit
Emgin et al. (2024) (11)	Turkey	2023	P	X	578 (M/F 326/252) 68.09 ± 16.62 years	CV	MR (28 days)	OR	Per unit
Fong et al. (2022) (26)	China	2014–2015	R	X	52,107 (M/F 28513/23594) 67 [55, 77] years	CV	MR (Hospital)	OR	Extreme quintiles
Furushima et al. (2021) (47)	Japan	2018–2019	P	X	40 (M/F 35/5) 70 [61, 77] years	MAGE	MR (90 days); ICU-free days	OR; β	Per unit
Gerbaud et al. (2022) (42)	France	2015–2016	R	C	392 (M/F 271/121) 73 ± 10.2 years	SD	Ad_C_	HR	High vs. low
Gunawan et al. (2025) (48)	Indonesia	2022–2024	R	M	233 (M/F 123/110) 60.49 ± 12.04 years	MAGE	MR (30 days)	OR	High vs. low
Hanna et al. (2021) (51)	Sweden/Australia	2012–2016	R	X	2,305 (M/F 1530/775)	GLI	MR (Hospital)	OR	High vs. low
Hartmann et al. (2022) (58)	Germany	2020–2021	R	X	106 (M/F 72/34) 63 [57, 71] years	DGV	MR (ICU)	HR	Per unit
Hermanides et al. (2010) (7)	Netherlands	2004–2007	R	X	5,728 (M/F 3757/1971) 65 ± 13 years	MAG	MR (ICU and hospital)	OR	Extreme quartiles
Hoang et al. (2024) (31)	USA	2020	R	B	112	SD; CV; MAGE; J-index	Composite complications including infection	OR	Per unit
Kim et al. (2022) (32)	South Korea	2018–2019	R	X	282 60.6 years	CV	MR (28 days) LOS (ICU)	OR	Per unit
Krinsley et al. (2020) (33)	USA	2011–2019	R	X	5,567	CV	MR (Hospital)	OR	Extreme tertiles
Kurtz et al. (2014) (43)	USA	2006–2009	R	N	28 (M/F 9/19) 54 [41, 61] years	SD	Ad_N_ (Cerebral metabolic distress); MR (Hospital)	OR	Per unit
Lanspa et al. (2014) (34)	USA	2006–2012	R	X	6,101 (M/F 3630/2471) 65 [53, 75] years	CV	MR (30 days)	OR	Per 10 unit
Lazzeri et al. (2020) (35)	Italy	2016–2018	P	T	252 (M/F 176/76)	SD; CV	MR (ICU)	OR	Per unit
Lazzeri et al. (2014) (44)	Italy	2012–2013	R	C	247	SD; MAGC	MR (Follow-up)	HR	Per unit
Li et al. (2019) (57)	China	2011–2013	R	C	137 (M/F 93/44) 64.45 ± 9.22 years	MODD	Ad_C_ (Arrhythmia)	OR	High vs. low
Liu et al. (2022) (52)	China	2017–2021	R	X	238 (M/F 143/95) 69.9 ± 12.6 years	GLI	DIC	sHR	Per unit
Ma et al. (2022) (36)	China	2019–2020	R	X	958 (M/F 613/345) 62.48 ± 17.86 years	CV	MR (Hospital)	OR	Per unit
Okazaki et al. (2018) (45)	Japan	2009–2015	R	N	122 (M/F 35/87) 61.5 ± 16.7 years	SD	Ad_N_	OR	Per unit
Okazaki et al. (2022) (55)	Japan	2020–2021	R	E	328 (M/F 216/112) 72 [61, 79] years	TIR	MR (Hospital and 28 days)	HR	High vs. low
Réa et al. (2023) (39)	Brazil	2020	R	X	841 (M/F 371/470) 61 ± 16.6 years	CV	MR (30 days)	OR	Per unit
Sadan et al. (2020) (59)	USA	2002–2016	R	N	2,451 (M/F 703/1748) 53 ± 14 years	ACACP; MCACP	MR (Hospital)	OR	Per unit
Sechterberger et al. (2013) (56)	Netherlands	2004–2011	R	X	10,320 (M/F 6836/3484) 65 ± 13 years	MAG	MR (ICU)	OR	Extreme quartiles
Sundarsingh et al. (2023) (37)	India	2017–2018	P	X	100 (M/F 54/46) 55 [41.25, 65] years	SD; CV; GLI; TIR	MR (28 day); Infection (bloodstream)	OR	High vs. low
Todi and Bhattacharya (2014) (46)	India	2009	R	X	2,208 (M/F 1302/906) 61 ± 16.71 years	SD; GLI	MR (ICU)	OR	Extreme quartiles; High vs. low
Yao et al. (2023) (38)	China	2020–2022	R	X	165	CV	PICS	OR	Per unit
Zhu et al. (2025) (49)	China	2008–2019	R	X	13,852 (M/F 8810/5042) 67 [57, 76] years	MAGE	MR (ICU, hospital, and 28 days)	HR	Extreme quartiles; Per SD
Zuo et al. (2012) (53)	China	2005–2010	R	M	294 (M/F 198/96) 51.1 ± 13.5 years	GLI	MR (ICU and hospital)	OR	Extreme quartiles

^†^R, retrospective; P, prospective; X, mixed ICU; C, cardiac ICU; N, neurological ICU; M, medical ICU; B, burn ICU; T, trauma ICU; E, emergency ICU.

^§^Sex breakdown (M/F) and age are included when available. Age is expressed as mean ± standard deviation or median [P25, P75].

^¶^GV metrics: SD, standard deviation; CV, coefficient of variation; MAG, mean absolute glucose; MAGE, mean amplitude of glycemic excursions; GLI, glycemic lability index; J-index, Jensen index; MODD, mean of daily differences; TIR, time in range; MSSD, mean of squared successive differences; MAGC, mean absolute glucose change per hour; DGV, daily glycemic variability; ACACP, average consecutive absolute change percentage; MCACP, median consecutive absolute change percentage.

^⸸^Outcome measures: MR, mortality rate; LOS, length of stay; Ad_N_, neurological adverse events, Ad_C_, cardiovascular adverse events; DIC, disseminated intravascular coagulation; AKI, acute kidney injury; PICS, persistent inflammation, immunosuppression, and catabolism syndrome. Effect estimates: OR, odds ratio; HR, hazard ratio, sHR, sub-distribution hazard ratio; β, linear regression coefficient.

The most commonly used GV metrics were CV [15 studies (11, 26–39)], SD [11 studies (27, 31, 35, 37, 40–46)], MAGE [6 studies (27, 28, 31, 47–49)], and glycemic lability index (GLI) [6 studies (37, 46, 50–53)]. Less frequently reported metrics included time in range [TIR, 3 studies (37, 54, 55)], MAG [2 studies (7, 56)], the mean of daily differences [MODD, 1 study (57)], mean absolute glucose change per hour [MAGC, 1 study (44)], and Jensen Index [J-index, 1 study (31)]. Three other infrequently used metrics were also included (58, 59).

In terms of outcome measures, 28 studies reported mortality-related outcomes, including ICU mortality [10 studies (7, 30, 35, 40, 46, 49, 50, 53, 56, 58)], hospital mortality [12 studies (7, 26, 33, 36, 40, 43, 49, 51, 53–55, 59)], 28/30-day mortality [9 studies (11, 28, 32, 34, 37, 39, 48, 49, 55)], 90-day mortality [2 studies (27, 47)], and follow-up mortality [1 study (44)]. Non-mortality outcomes included infection [4 studies (31, 37, 41, 50)], length of stay measures [4 studies (29, 32, 41, 47)], free-day measures [2 studies (29, 47)], neurological adverse events [2 studies (43, 45)], cardiovascular adverse events [2 studies (42, 57)], readmission [1 study (41)], disseminated intravascular coagulation [DIC, 1 study (52)], acute kidney injury [AKI, 1 study (41)], and persistent inflammation, immunosuppression, and catabolism syndrome [PICS, 1 study (38)].

### 3.2 Correlation of GV with mortality measure

Due to differences in GV metrics, outcomes, and adjustment status, only 25 of the 36 studies with suitable data for quantitative synthesis, were included in the meta-analysis, and 11 studies were included for qualitative synthesis.

#### 3.2.1 ICU mortality

Ten studies (7, 30, 35, 40, 46, 49, 50, 53, 56, 58) reported on GV and ICU mortality. Meta-analyses were conducted between CV (2 studies), SD (2 studies), MAG (2 studies), GLI (2 studies) and ICU mortality, respectively.

**Figure 2:**
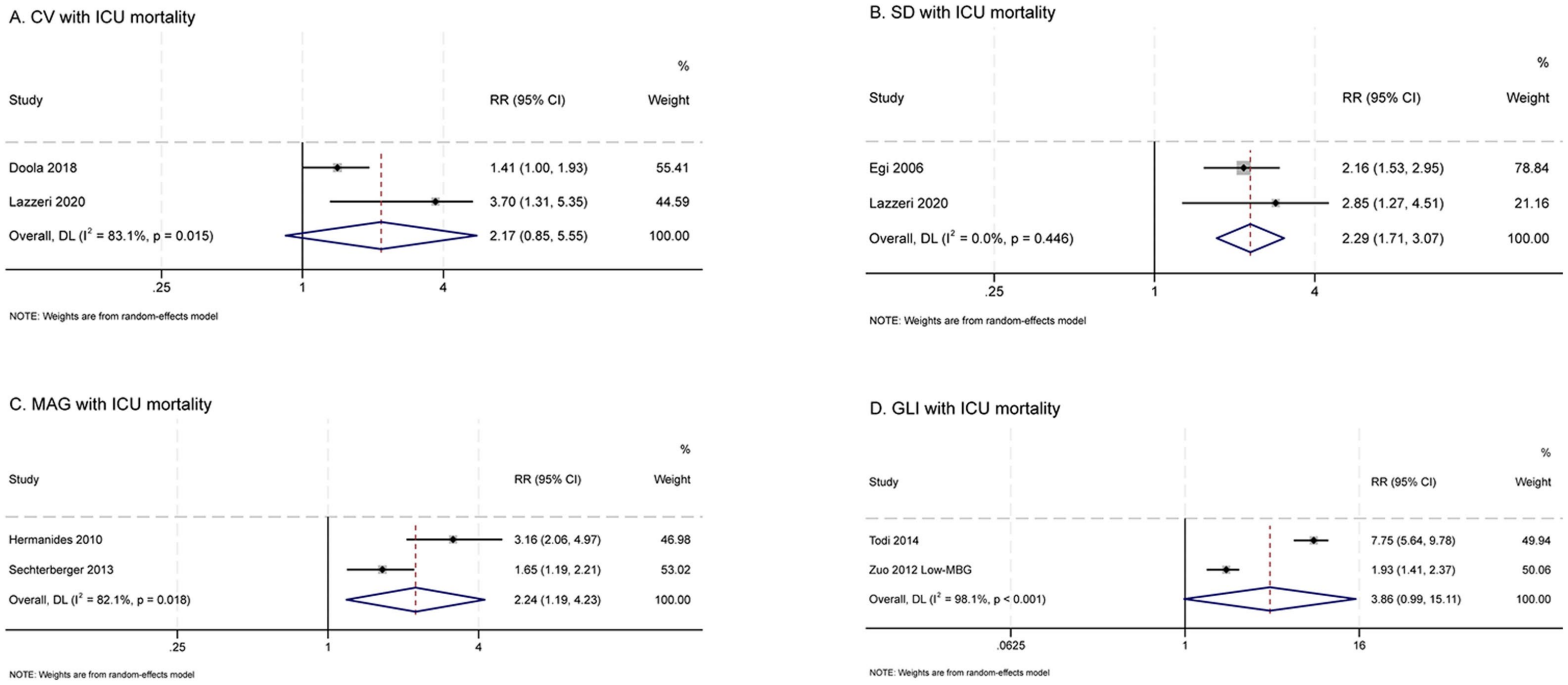
Forest plots for the associations of glycemic variability (extreme quartiles) with ICU mortality using random-effects meta-analysis. **(A)** CV with ICU mortality. **(B)** SD with ICU mortality. **(C)** MAG with ICU mortality. **(D)** GLI with ICU mortality.

Two studies (30, 35) evaluated CV; their pooled analysis suggested a non-significant trend toward increased mortality risk (adjusted RR = 2.17, 95% CI 0.85–5.55) (Figure 2A). Heterogeneity was high (*I*^2^ = 83.1%) and the certainty of evidence was very low (Table 2). The supplementary validation using original estimates is presented in Supplementary Figure 1.

**Table 2:** GRADE summary of findings for short-term glycemic variability and adverse ICU outcomes in critically ill patients.

Outcomes	No. of studies	Participants (n)	Effect estimate (95% CI)	Certainty of evidence (GRADE)
CV with 28/30-day mortality	6	8,354	1.34 (1.10, 1.63)	Low^a,b^
MAGE with 28/30-day mortality	2	14,304	2.05 (1.52, 2.77)	Low^a,c^
MAGE with 90-day mortality	2	198	2.90 (1.96, 4.30)	Low^a,c^
CV with ICU mortality	2	1,011	2.17 (0.85, 5.55)	Very low^a,b,c^
SD with ICU mortality	2	7,301	2.29 (1.71, 3.07)	Low^a,c^
MAG with ICU mortality	2	16,048	2.24 (1.19, 4.23)	Very low^a,b,c^
GLI with ICU mortality	2	2,502	3.86 (0.99, 15.11)	Very low^a,b,c^
CV with hospital mortality	3	58,632	1.39 (1.05, 1.85)	Low^a,c^
SD with hospital mortality	2	7,077	2.26 (1.19, 4.30)	Very low^a,b,c^
SD with infection	2	982	1.02 (1.01, 1.04)	Low^a,c^
SD with neurological adverse events	2	150	1.23 (0.91, 1.66)	Very low^a,b,c^

The following footnotes describe the general reasons for downgrading the certainty of evidence across outcomes.

^a^Evidence certainty was downgraded one level due to risk of bias (predominantly retrospective observational studies with potential residual confounding).

^b^Evidence certainty was downgraded one level due to inconsistency (substantial heterogeneity across studies; *I*^2^ > 50%).

^c^Evidence certainty was downgraded one level due to imprecision (only two studies were included).

Two studies (35, 40) reported on SD; the pooled analysis linked increased SD to ICU mortality (adjusted RR = 2.29, 95% CI 1.71–3.07) (Figure 2B), but evidence certainty was low (Table 2). The supplementary validation using original estimates is presented in Supplementary Figure 2.

Two studies (7, 56) evaluated MAG; while a significant association was observed (adjusted RR = 2.24, 95% CI 1.19–4.23), heterogeneity was substantial (*I*^2^ = 82.1%) (Figure 2C) and evidence certainty was very low (Table 2). The supplementary validation using original estimates is presented in Supplementary Figure 3.

Three studies (46, 50, 53) reported on GLI and ICU mortality, and two of them (46, 53) were included in a meta-analysis. The pooled results indicated a trend toward increased mortality risk (unadjusted RR = 3.86, 95% CI 0.99–15.11), but the heterogeneity was high (*I*^2^ = 98.1%) (Figure 2D) and evidence certainty was very low (Table 2). The supplementary validation using original estimates with harmonized exposure is presented in Supplementary Figure 4.

In addition, Hartmann et al. (58) found that each unit increase in daily glycemic variability (DGV) was associated with increased ICU mortality risk (adjusted HR = 1.02, 95% CI 1.01–1.03), and Zhu et al. (49) reported higher ICU mortality risk for patients in the highest MAGE quartile versus the lowest (adjusted HR = 3.59, 95% CI 2.99–4.31).

#### 3.2.2 Hospital mortality

Twelve studies (7, 26, 33, 36, 40, 43, 49, 51, 53–55, 59) reported on GV and hospital mortality. Meta-analyses were conducted between CV (3 studies), SD (2 studies) and hospital mortality, respectively.

**Figure 3:**
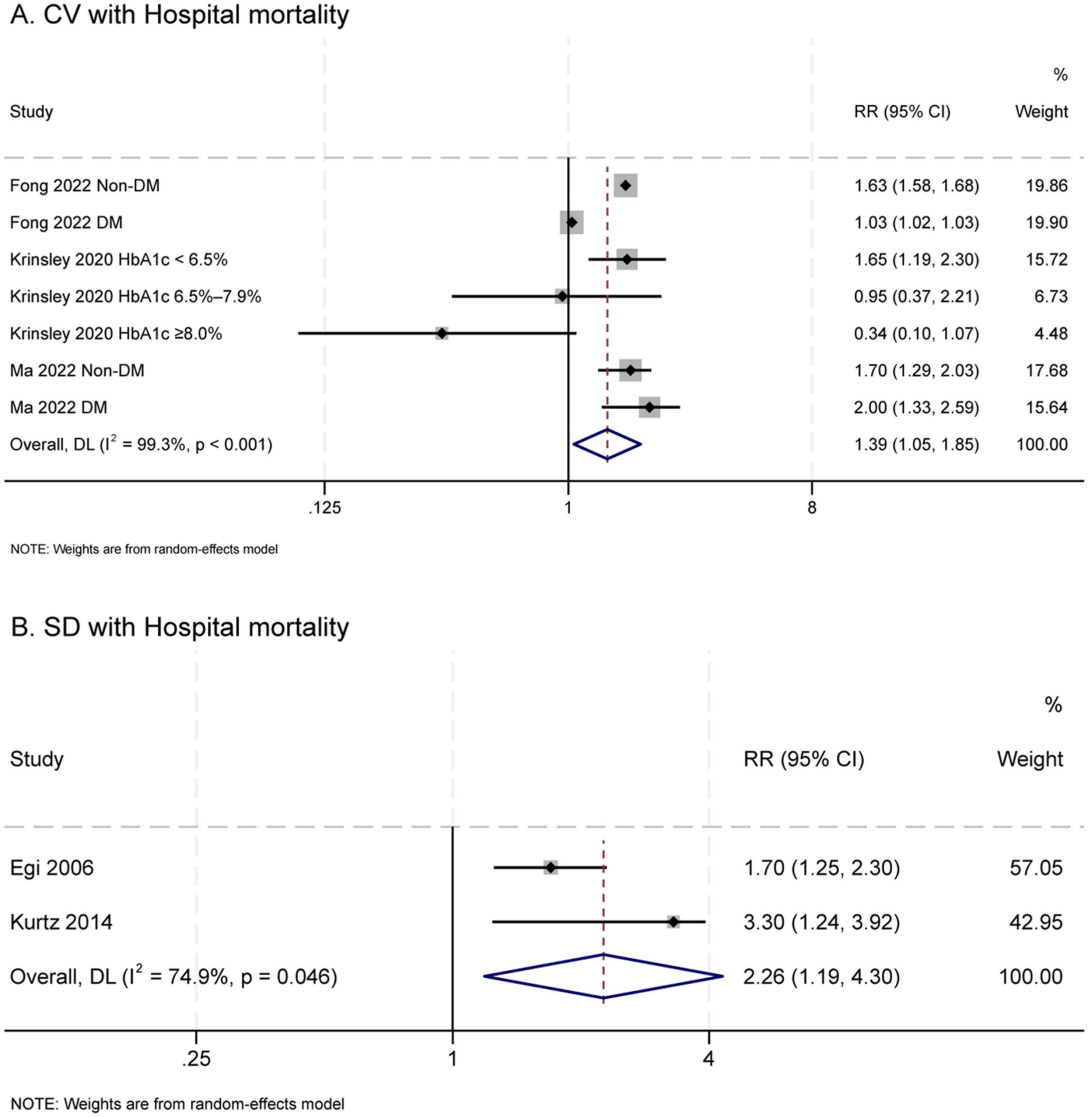
Forest plots for the associations of glycemic variability (extreme quartiles) with hospital mortality using random-effects meta-analysis. **(A)** CV with Hospital mortality. **(B)** SD with Hospital mortality.

Three studies (26, 33, 36) focused on CV; their pooled results linked higher CV to increased hospital mortality risk (adjusted RR = 1.39, 95% CI 1.05–1.85), but heterogeneity was high (*I*^2^ = 99.3%) (Figure 3A) and evidence certainty was low (Table 2). The supplementary validation using original estimates with harmonized exposure contrasts, together with sensitivity analyses, are presented in Supplementary Figures 5, 11.

Two studies (40, 43) evaluated SD, and their pooled analysis associated higher SD with increased hospital mortality risk (adjusted RR = 2.26, 95% CI 1.19–4.30), with high heterogeneity (*I*^2^ = 74.9%) (Figure 3B) and very low evidence certainty (Table 2). The supplementary validation using original estimates is presented in Supplementary Figure 6.

The remaining seven studies (7, 49, 51, 53–55, 59) were excluded from quantitative synthesis due to differences in GV metrics and effect estimate reporting.

Among these, two studies reported that higher GLI was associated with increased hospital mortality, including Hanna et al. (51) (adjusted OR = 1.60, 95% CI 1.19–2.15) and Zuo et al. (53) (unadjusted OR = 3.57, 95% CI 1.81–7.06).

Two studies (54, 55) examined TIR-related metrics. Ammar et al. (54) linked higher TIR to lower hospital mortality risk (adjusted OR = 0.52, 95% CI 0.27–0.97). Okazaki et al. (55) found that hospital mortality risk was significantly associated with relative normoglycemia TIR (defined as 70–140% of HbA1c-derived average; adjusted OR = 0.16, 95% CI 0.06–0.43) but not absolute normoglycemia TIR (70–140 mg/dL; adjusted OR = 0.44, 95% CI 0.15–1.23).

Three studies (7, 49, 59) examined other GV metrics: MAG, MAGE, average consecutive absolute percentage change (ACACP), and median consecutive absolute percentage change (MCACP). Hermanides et al. (7) found that the highest MAG quartile had a higher mortality risk than the lowest (adjusted OR = 2.80, 95% CI 2.00–3.90). Sadan et al. (59) detected elevated hospital mortality for each unit increase in ACACP (adjusted OR = 5.18, 95% CI 1.37–19.82) and MCACP (adjusted OR = 8.82, 95% CI 1.80–43.56). Zhu et al. (49) showed that the highest MAGE quartile was associated with higher hospital mortality compared to the lowest (adjusted HR = 3.43, 95% CI 2.92–4.02), and a 1-SD increase in MAGE was also associated with mortality (adjusted HR = 1.31, 95% CI 1.27–1.35).

#### 3.2.3 28/30-day mortality

Nine studies (11, 28, 32, 34, 37, 39, 48, 49, 55) reported on the association between GV and 28/30-day mortality. Meta-analyses were conducted between CV (6 studies), MAGE (2 studies) and 28/30-day mortality, respectively.

**Figure 4:**
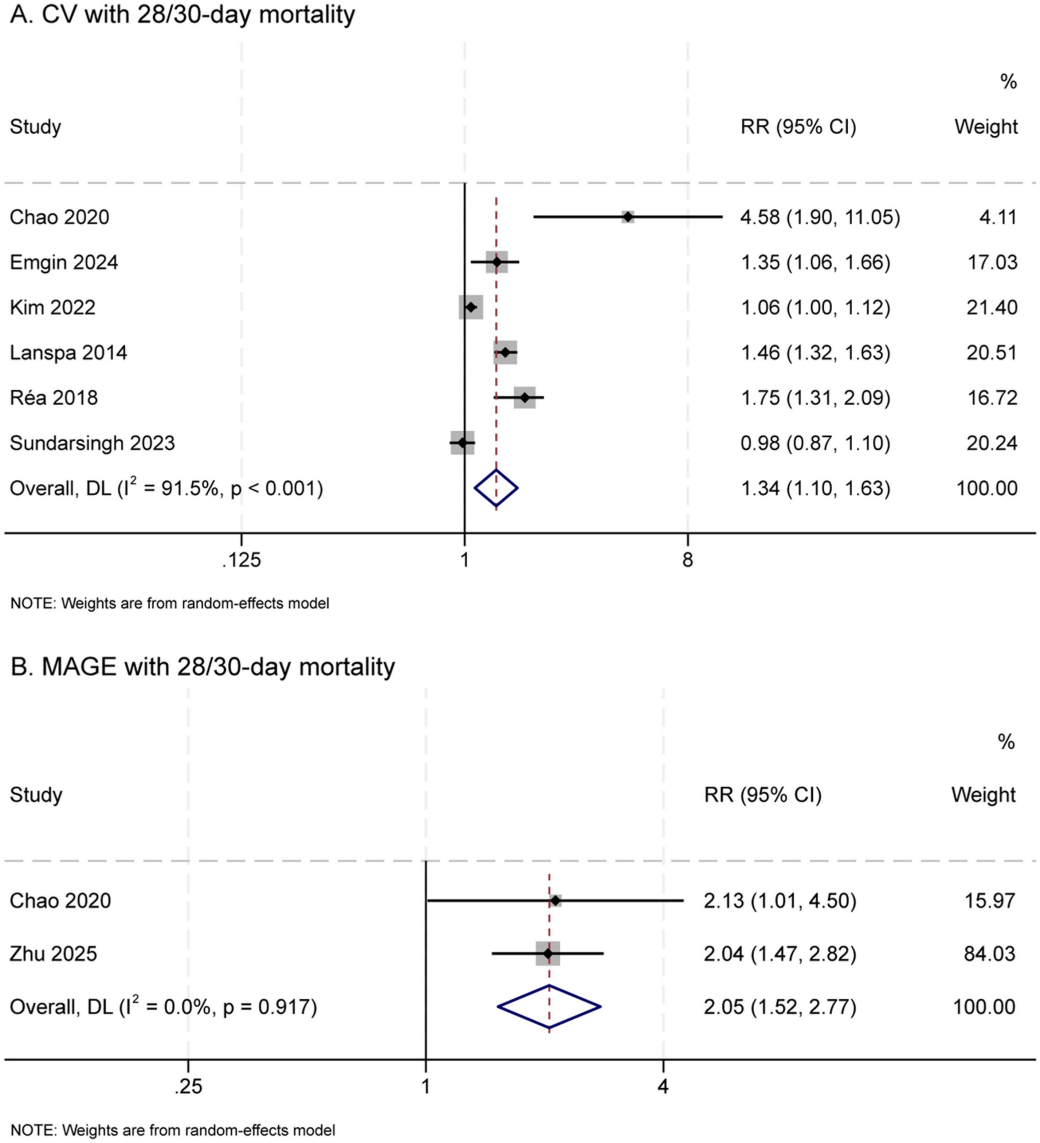
Forest plots for the associations of glycemic variability (extreme quartiles) with 28/30-day mortality using random-effects meta-analysis. **(A)** CV with 28/30-day mortality. **(B)** MAGE with 28/30-day mortality.

Six studies (11, 28, 32, 34, 37, 39) evaluated CV and were included in a meta-analysis. Pooled results linked higher CV to increased risk of 28/30-day mortality (adjusted RR = 1.34, 95% CI 1.10–1.63), with substantial heterogeneity (*I*^2^ = 91.5%) (Figure 4A). The certainty of evidence was rated as low (Table 2). The supplementary validation using original estimates with harmonized exposure contrasts, together with sensitivity analyses, are presented in Supplementary Figures 7, 8, 12.

Three studies (28, 48, 49) examined MAGE and 28/30-day mortality, and two of them were included in a meta-analysis. When the highest and lowest quartiles were compared, higher MAGE was associated with elevated mortality (adjusted RR = 2.05, 95% CI 1.52–2.77); there was no heterogeneity (*I*^2^ = 0.0%) (Figure 4B), but the evidence certainty was low (Table 2). The supplementary validation using original estimates with harmonized exposure is presented in Supplementary Figure 9.

Two studies (37, 55) evaluated TIR and 28/30-day mortality. Okazaki et al. (55) found that higher TIR of relative normoglycemia was associated with a lower 28-day mortality risk (adjusted HR = 0.21, 95% CI 0.08–0.58). Sundarsingh et al. (37) observed a non-significant trend toward a lower 28-day mortality risk with higher TIR (adjusted OR = 0.72, 95% CI 0.31–1.66).

#### 3.2.4 90-day mortality

**Figure 5:**
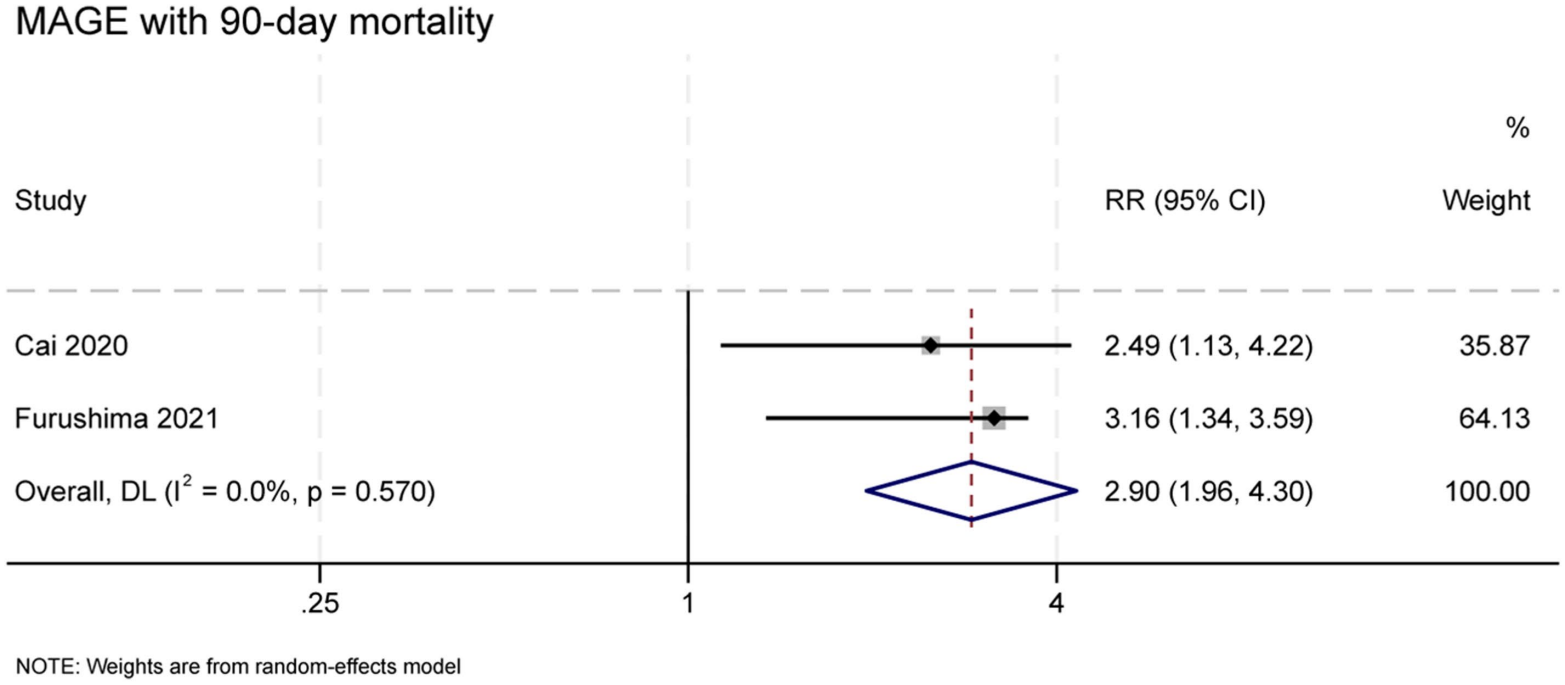
Forest plots for the associations of glycemic variability (extreme quartiles) with 90-day mortality using random-effects meta-analysis.

Two studies (27, 47) reported on MAGE and 90-day mortality. A meta-analysis of these two studies indicated that patients in the highest MAGE quartile (Q4) had a higher risk of 90-day mortality than those in the lowest (Q1) (adjusted RR = 2.90, 95% CI 1.96–4.30, *p* < 0.001) (Figure 5), but the evidence certainty was low (Table 2). The supplementary validation using original estimates is presented in Supplementary Figure 10.

### 3.3 Correlation of GV with non-mortality outcome measures

#### 3.3.1 Infection

**Figure 6:**
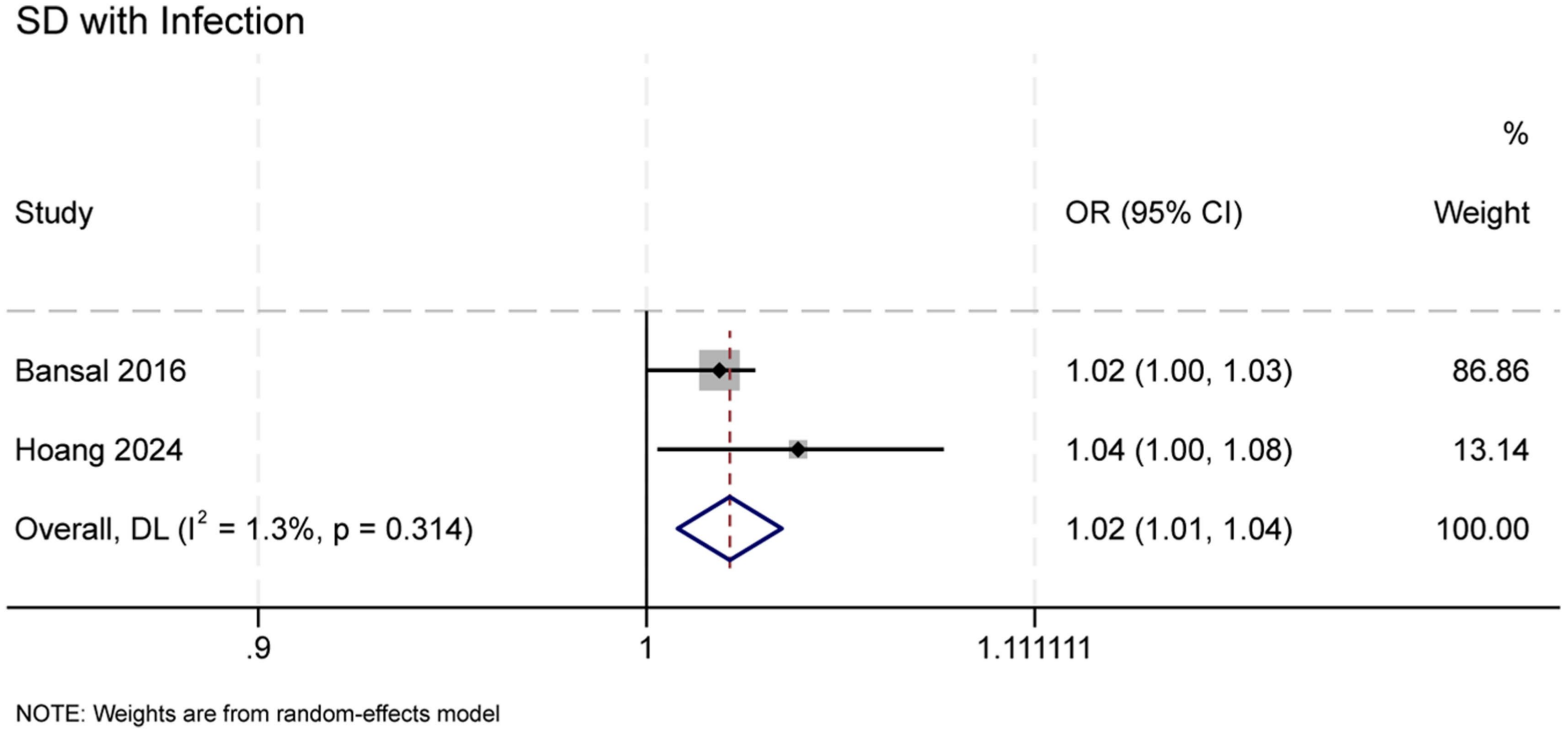
Forest plots for the associations of glycemic variability (per unit increase) with infection using random-effects meta-analysis.

Four studies (31, 37, 41, 50) reported on GV and infection outcomes. Three studies (31, 37, 41) evaluated the relationship between SD and infection, and two of them (31, 41) were included in a meta-analysis. The pooled analysis showed that each unit increase in SD was associated with infection (adjusted OR = 1.02, 95% CI 1.01–1.04); the heterogeneity was low (*I*^2^ = 1.3%) (Figure 6) but the evidence certainty was also low (Table 2).

Of the two studies excluded from meta-analysis, Donati et al. (50) reported that higher GLI was associated with ICU-acquired infection (adjusted OR = 2.27, 95% CI 1.64–3.16). Sundarsingh et al. (37) found that lower infection risk was associated with higher TIR (unadjusted OR = 0.31, 95% CI 0.13–0.74, *p* = 0.009) but no association was observed for SD, CV, or GLI.

#### 3.3.2 Neurological adverse events

**Figure 7:**
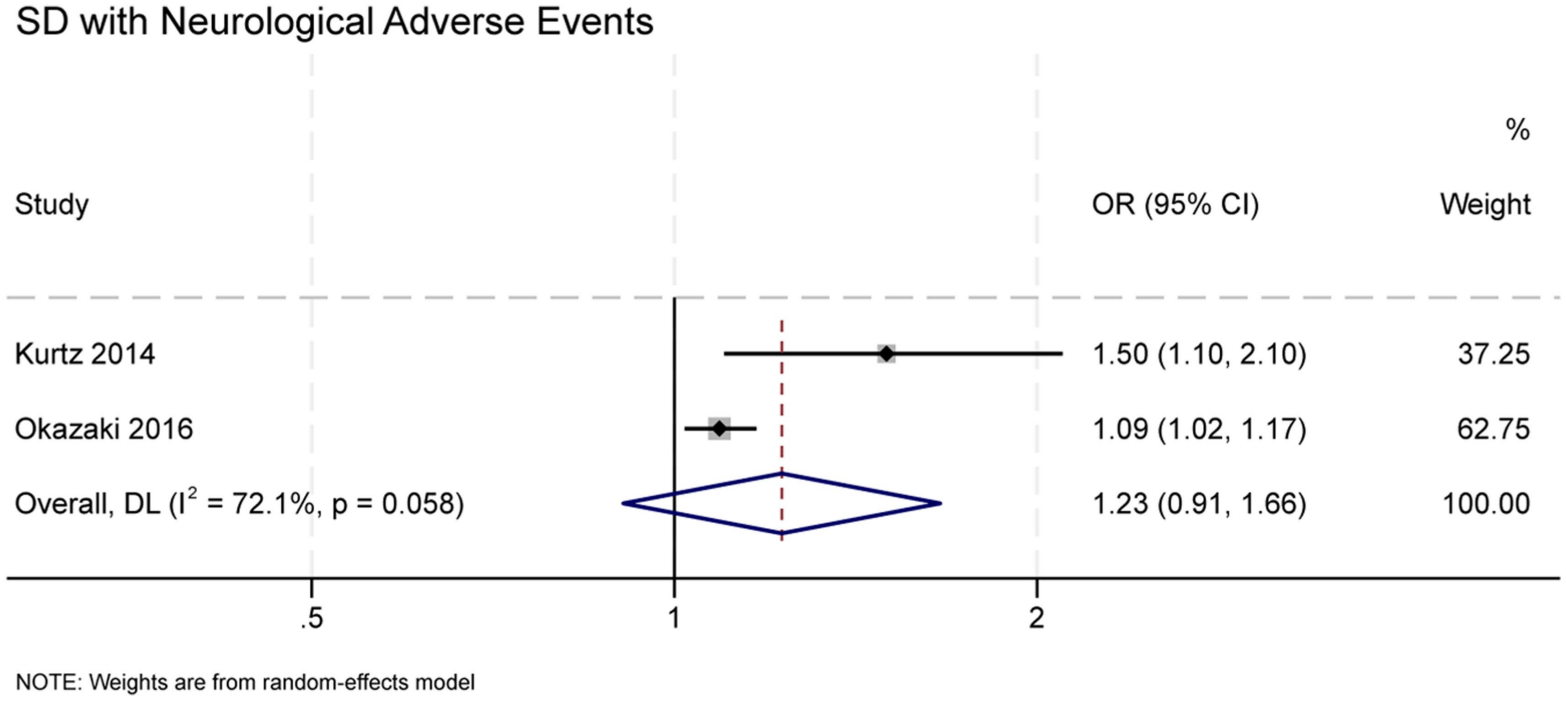
Forest plots for the associations of glycemic variability (per unit increase) with neurological adverse events using random-effects meta-analysis.

Two studies (43, 45) reported on SD and neurological adverse events. The pooled analysis of these two studies indicated that a 1-unit increase in SD was not associated with neurological adverse events (adjusted OR = 1.23, 95% CI 0.91–1.66); the heterogeneity (*I*^2^ = 72.1%) was substantial (Figure 7) and the evidence certainty was very low (Table 2).

## 4 Discussion

### 4.1 Heterogeneity analysis and methodological considerations

We reviewed 36 observational studies with moderate-to-high methodological quality. The certainty of evidence was rated as low initially for all studies and further downgraded for some outcomes due to heterogeneity (*I*^2^ > 50%). Therefore, random-effects models were used, and the analyses were stratified by GV metric and outcome type. Although substantial heterogeneity remained in some analyses, the overall consistency in the direction of effects across studies to some extent supports the robustness of the findings and their potential clinical significance. In addition, leave-one-out sensitivity analyses showed that no single study was the main driver of heterogeneity. This suggests that the substantial heterogeneities (*I*^2^ > 75% for some meta-analyses) likely reflect genuine clinical and methodological diversity rather than fundamental inconsistencies in effect. And the sources of heterogeneity may include variations in patient populations, GV metric definitions, monitoring frequency, sampling density, measurement time window, covariate adjustment, etc. In critical care, such variability is plausible and matches real-world practice (60). Additionally, the included studies span a long period (2006–2025), during which ICU glycemic management has evolved. The publication of the NICE-SUGAR trial in 2009 marked a key turning point (61), shifting practice from intensive to more moderate glycemic targets with greater emphasis on hypoglycemia avoidance, as well as influencing monitoring frequency and insulin strategies, which may have contributed to the observed heterogeneity.

Furthermore, differences in exposure contrasts and the diversity of effect measure formats across studies substantially increase the difficulty of cross-study combination. To improve the feasibility of data combination, we standardized all effect estimates to a unified metric. This approach has been used in previous several studies (8, 62, 63), and further supported by methodological research as a means of achieving effective data integration (21). In our research, the standardized effect direction were consistent with the additional analysis using original estimates, which further indicates that these transformations have limited influence on the direction of effects (64). Accordingly, the results should not be interpreted as precise effect estimates, but rather as indicators reflecting the overall direction and relative effect of the associations.

### 4.2 Clinical implications and potential mechanisms of short-term GV

We comprehensively evaluated the associations between multiple short-term GV metrics and adverse ICU outcomes. Overall, elevated short-term GV significantly predicted unfavorable outcomes. As to the associations between GV and mortality, we analyzed outcomes at specific time intervals rather than combining the results from different mortality time points as previous studies have done (17). Specifically, MAGE showed stable associations with 28/30-day and 90-day mortality, whereas CV and SD predicted 28/30-day, ICU, and in-hospital mortality. These findings suggest that short-term GV serves as a key prognostic marker complementary to mean glucose, consistent with prior clinical evidence in critically ill populations (17, 65). Additionally, we captured the growing evidence base and performed robust quantitative syntheses of underrepresented metrics, finding that both MAG and GLI are associated with ICU mortality. We also extended the evidence to non-mortality outcomes and identified a significant association between SD and infection risk. In contrast, evidence regarding the association between SD and neurological adverse events remains statistically non-significant, which highlights the need for further high-quality studies.

While mean glucose remains a key reference, the independent associations of GV metrics with mortality and infection observed in our study underscore the clinical relevance of glycemic fluctuation itself (66). These findings imply that in the ICU setting, short-term GV may serve as an important complement to mean glucose, providing additional value for risk stratification and glycemic management (67). However, the strength of the associations between GV metrics and ICU outcomes varied across these metrics, with some showing relatively weak associations. This variation in association strength identified in our study may stem from two key factors. First, the number of studies for each metric–outcome pair is highly variable, leading to differences in statistical power (68). Second, different GV metrics are derived from distinct mathematical approaches and may capture different fluctuation patterns (69). For example, SD measures the absolute dispersion of glucose values around the mean and thereby reflects the total magnitude of glycemic deviation, whereas CV normalizes for average glucose levels by expressing the SD as a percentage of the mean, thus providing a measure of relative variability that allows for comparison across different glycemic ranges (40, 70). In contrast, MAGE includes only glucose peak-to-nadir excursions whose amplitudes exceed one SD; by specifically targeting large-amplitude glucose fluctuations, it may more accurately reflect the acute metabolic instability and cellular stress seen in critically ill patients (12). Collectively, these metrics provide complementary information and should be selected for specific clinical or research goals, in combination with other glycemic indicators, to allow comprehensive evaluation of glycemic status in clinical settings (71).

Based on previous evidence (6, 72), we speculate that short-term GV may contribute to adverse clinical outcomes through a cascading pathophysiological sequence. Rapid fluctuations in blood glucose are thought to be associated with increased production of reactive oxygen species (12), which may enhance oxidative stress and activate systemic inflammatory signaling (72). This inflammatory and oxidative milieu has been linked to endothelial dysfunction and microcirculatory impairment, potentially leading to organ hypoperfusion (14). Together, these vascular and metabolic disturbances may compromise immune function, thereby increasing susceptibility to infection and contributing to the development of multiple organ dysfunction (73, 74). Furthermore, frequent shifts between hyperglycemia and hypoglycemia disrupt neuroendocrine homeostasis, which alters stress hormone secretion and insulin sensitivity, thus creating a vicious cycle of metabolic dysregulation (75). Because ICU patients are physiologically unstable, acute fluctuations captured by short-term GV may reflect disease severity and treatment response more sensitively than mean glucose (11, 66).

### 4.3 Limitations

Although this study involved systematic literature searching, data processing, and a relatively large sample size, several limitations should be noted. First, only English-language studies were included, which may introduce language bias. Second, some outcomes showed substantial heterogeneity, and the small number of included studies precluded both planned subgroup analyses for exploring heterogeneity sources and Egger’s test for publication bias assessment. Third, some GV-outcomes lacked sufficient data for quantitative synthesis. In addition, studies using only simple range-based measures were excluded; although this improved methodological comparability, it may have led to the omission of potentially informative evidence. Finally, as all included studies were observational, residual confounding cannot be fully excluded.

Given these limitations, the findings should be interpreted with caution. Future well-designed prospective studies with larger sample sizes, standardized GV assessment, and unified outcome definitions are needed, along with multi-database data to validate robustness. In addition, further studies are needed to evaluate the associations between a broader range of GV metrics, including simple range-based indicators, and adverse ICU outcomes in critically ill patients, and to assess their predictive performance across clinical settings through subgroup analyses.

## 5 Conclusion

In summary, in critically ill patients, short-term GV is associated with increased risks of mortality (28/30-day, 90-day, ICU, and in-hospital) and infection. These findings suggest that incorporating short-term GV metrics can improve prognostic risk stratification beyond monitoring mean glucose alone. Further high-quality studies are needed to determine the optimal clinical application of GV metrics for risk assessment and management.

## Data Availability

The datasets presented in this study can be found in online repositories. The names of the repository/repositories and accession number(s) can be found in the article/Supplementary material.
