# Supplementary material for: Short-term glycemic variability and the risk of adverse ICU outcomes in critically ill patients: a systematic review and meta-analysis
Source: Front Nutr. 2026 Jun 9;13:1808757. doi: 10.3389/fnut.2026.1808757 (PMC13286760; doi:10.3389/fnut.2026.1808757)
Supplement: Supplementary file 1 [file Table_1.docx]

Supplementary Material

# **Supplementary Table 1.** PRISMA 2020 checklist for systematic reviews and meta-analyses

| Section and Topic | Item # | Checklist item | Location where item is reported |
| --- | --- | --- | --- |
| TITLE | | |  |
| Title | 1 | Identify the report as a systematic review. | Title |
| ABSTRACT | | |  |
| Abstract | 2 | See the PRISMA 2020 for Abstracts checklist. | Abstract |
| INTRODUCTION | | |  |
| Rationale | 3 | Describe the rationale for the review in the context of existing knowledge. | Introduction |
| Objectives | 4 | Provide an explicit statement of the objective(s) or question(s) the review addresses. | Introduction |
| METHODS | | |  |
| Eligibility criteria | 5 | Specify the inclusion and exclusion criteria for the review and how studies were grouped for the syntheses. | Methods – Selection criteria |
| Information sources | 6 | Specify all databases, registers, websites, organisations, reference lists and other sources searched or consulted to identify studies. Specify the date when each source was last searched or consulted. | Methods – Search strategy |
| Search strategy | 7 | Present the full search strategies for all databases, registers and websites, including any filters and limits used. | Supplementary Table 2 |
| Selection process | 8 | Specify the methods used to decide whether a study met the inclusion criteria of the review, including how many reviewers screened each record and each report retrieved, whether they worked independently, and if applicable, details of automation tools used in the process. | Methods – Study selection |
| Data collection process | 9 | Specify the methods used to collect data from reports, including how many reviewers collected data from each report, whether they worked independently, any processes for obtaining or confirming data from study investigators, and if applicable, details of automation tools used in the process. | Methods – Data extraction |
| Data items | 10a | List and define all outcomes for which data were sought. Specify whether all results that were compatible with each outcome domain in each study were sought (e.g. for all measures, time points, analyses), and if not, the methods used to decide which results to collect. | Methods – Selection criteria |
|  | 10b | List and define all other variables for which data were sought (e.g. participant and intervention characteristics, funding sources). Describe any assumptions made about any missing or unclear information. | Methods – Data extraction; Supplementary Table 3 |
| Study risk of bias assessment | 11 | Specify the methods used to assess risk of bias in the included studies, including details of the tool(s) used, how many reviewers assessed each study and whether they worked independently, and if applicable, details of automation tools used in the process. | Methods – Study quality assessment and risk of bias |
| Effect measures | 12 | Specify for each outcome the effect measure(s) (e.g. risk ratio, mean difference) used in the synthesis or presentation of results. | Methods – Data extraction; Supplementary Methods |
| Synthesis methods | 13a | Describe the processes used to decide which studies were eligible for each synthesis (e.g. tabulating the study intervention characteristics and comparing against the planned groups for each synthesis (item #5)). | Methods – Statistical analysis |
|  | 13b | Describe any methods required to prepare the data for presentation or synthesis, such as handling of missing summary statistics, or data conversions. | Supplementary Methods |
|  | 13c | Describe any methods used to tabulate or visually display results of individual studies and syntheses. | Methods – Statistical analysis |
|  | 13d | Describe any methods used to synthesize results and provide a rationale for the choice(s). If meta-analysis was performed, describe the model(s), method(s) to identify the presence and extent of statistical heterogeneity, and software package(s) used. | Methods – Statistical analysis |
|  | 13e | Describe any methods used to explore possible causes of heterogeneity among study results (e.g. subgroup analysis, meta-regression). | Methods – Statistical analysis |
|  | 13f | Describe any sensitivity analyses conducted to assess robustness of the synthesized results. | Methods – Statistical analysis |
| Reporting bias assessment | 14 | Describe any methods used to assess risk of bias due to missing results in a synthesis (arising from reporting biases). | Methods – Statistical analysis |
| Certainty assessment | 15 | Describe any methods used to assess certainty (or confidence) in the body of evidence for an outcome. | Methods – Statistical analysis |
| RESULTS | | |  |
| Study selection | 16a | Describe the results of the search and selection process, from the number of records identified in the search to the number of studies included in the review, ideally using a flow diagram. | Results – Study selection and characteristics; Figure 1 |
|  | 16b | Cite studies that might appear to meet the inclusion criteria, but which were excluded, and explain why they were excluded. | Supplementary Table 4 |
| Study characteristics | 17 | Cite each included study and present its characteristics. | Results – Study selection and characteristics; Table 1 |
| Risk of bias in studies | 18 | Present assessments of risk of bias for each included study. | Supplementary Table 5 |
| Results of individual studies | 19 | For all outcomes, present, for each study: (a) summary statistics for each group (where appropriate) and (b) an effect estimate and its precision (e.g. confidence/credible interval), ideally using structured tables or plots. | Results; Table 1; Figures 2–7; Supplementary Figures 1–12 |
| Results of syntheses | 20a | For each synthesis, briefly summarise the characteristics and risk of bias among contributing studies. | Results – Study selection and characteristic; Table 1; Supplementary Table 5 |
|  | 20b | Present results of all statistical syntheses conducted. If meta-analysis was done, present for each the summary estimate and its precision (e.g. confidence/credible interval) and measures of statistical heterogeneity. If comparing groups, describe the direction of the effect. | Results; Figures 2–7 |
|  | 20c | Present results of all investigations of possible causes of heterogeneity among study results. | Results – Correlation of GV with mortality measure |
|  | 20d | Present results of all sensitivity analyses conducted to assess the robustness of the synthesized results. | Supplementary Figures 11-12 |
| Reporting biases | 21 | Present assessments of risk of bias due to missing results (arising from reporting biases) for each synthesis assessed. | Not applicable |
| Certainty of evidence | 22 | Present assessments of certainty (or confidence) in the body of evidence for each outcome assessed. | Table 2 |
| DISCUSSION | | |  |
| Discussion | 23a | Provide a general interpretation of the results in the context of other evidence. | Discussion |
|  | 23b | Discuss any limitations of the evidence included in the review. | Limitations |
|  | 23c | Discuss any limitations of the review processes used. | Discussion; Limitations |
|  | 23d | Discuss implications of the results for practice, policy, and future research. | Discussion; Limitations |
| OTHER INFORMATION | | |  |
| Registration and protocol | 24a | Provide registration information for the review, including register name and registration number, or state that the review was not registered. | Methods |
|  | 24b | Indicate where the review protocol can be accessed, or state that a protocol was not prepared. | Not applicable |
|  | 24c | Describe and explain any amendments to information provided at registration or in the protocol. | Not applicable |
| Support | 25 | Describe sources of financial or non-financial support for the review, and the role of the funders or sponsors in the review. | Funding |
| Competing interests | 26 | Declare any competing interests of review authors. | Conflict of interest |
| Availability of data, code and other materials | 27 | Report which of the following are publicly available and where they can be found: template data collection forms; data extracted from included studies; data used for all analyses; analytic code; any other materials used in the review. | Data availability statement |

# **Supplementary Table 2.** Search strategy to identify studies reporting the associations of glycemic variability with ICU outcomes.

| **Database^§^** | **Search terms** | |
| --- | --- | --- |
| **PubMed** | #1 | Critical Illness[MeSH] OR critical illness[Title/Abstract] OR severe illness[Title/Abstract] OR life-threatening disease[Title/Abstract] OR life-threatening condition[Title/Abstract] OR critically ill patients[Title/Abstract] OR ICU patients[Title/Abstract] OR intensive care unit patients[Title/Abstract] |
|  | #2 | Critical Care Nursing[MeSH] OR critical care nursing[Title/Abstract] OR intensive care nursing[Title/Abstract] OR ICU nursing[Title/Abstract] OR critical care nurse[Title/Abstract] OR intensive care nurse[Title/Abstract] OR ICU nurse[Title/Abstract] |
|  | #3 | Critical Care[MeSH] OR intensive care[Title/Abstract] OR critical care medicine[Title/Abstract] OR intensive care medicine[Title/Abstract] OR critical care unit[Title/Abstract] OR intensive care unit[Title/Abstract] OR ICU[Title/Abstract] OR critical care management[Title/Abstract] |
|  | #4 | #1 OR #2 OR #3 |
|  | #5 | Glycated Hemoglobin[MeSH] OR glycated hemoglobin[Title/Abstract] OR glycosylated hemoglobin[Title/Abstract] OR HbA1c[Title/Abstract] OR A1c[Title/Abstract] OR glycemic[Title/Abstract] OR glycaemic[Title/Abstract] OR glycemia[Title/Abstract] OR glycaemia[Title/Abstract] |
|  | #6 | Blood Glucose[MeSH] OR blood glucose[Title/Abstract] OR plasma glucose[Title/Abstract] OR blood sugar[Title/Abstract] OR plasma sugar[Title/Abstract] OR FPG[Title/Abstract] OR PPG[Title/Abstract] |
|  | #7 | Search(#5 OR #6 ) |
|  | #8 | variability[Title/Abstract] OR variabilities[Title/Abstract] OR variation[Title/Abstract] OR variations[Title/Abstract] OR fluctuation[Title/Abstract] OR fluctuations[Title/Abstract] OR oscillation[Title/Abstract] OR oscillations[Title/Abstract] OR excursion[Title/Abstract] OR excursions[Title/Abstract] |
|  | #9 | Search (#7AND #8) |
|  | #10 | Prognosis[MeSH] OR prognosis[Title/Abstract] OR prognostic[Title/Abstract] OR prognostication[Title/Abstract] OR outcome prediction[Title/Abstract] OR clinical prognosis[Title/Abstract] OR disease prognosis[Title/Abstract] OR predictive outcome[Title/Abstract] |
|  | #11 | Mortality[MeSH] OR mortality[Title/Abstract] OR death rate[Title/Abstract] OR death rates[Title/Abstract] OR mortality rate[Title/Abstract] OR mortality rates[Title/Abstract] OR fatality[Title/Abstract] OR fatality rate[Title/Abstract] OR death ratio[Title/Abstract] |
|  | #12 | duration of therapy[Title/Abstract] OR length of stay[Title/Abstract] OR survival rate[Title/Abstract] OR therapy duration[Title/Abstract] OR duration of treatment[Title/Abstract] OR treatment duration[Title/Abstract] OR stay length[Title/Abstract] OR survival[Title/Abstract] OR incidence[Title/Abstract] OR outcomes[Title/Abstract] |
|  | #13 | #10 OR #11 OR #12 |
|  | #14 | #4AND #9AND#13 |
| **EMBASE** | 1 | 'critical illness'/exp |
|  | 2 | 'critical illness':ab,ti OR 'severe illness':ab,ti OR 'life-threatening disease':ab,ti OR 'life-threatening condition':ab,ti OR 'critically ill patients':ab,ti OR 'ICU patients':ab,ti OR 'intensive care unit patients':ab,ti |
|  | 3 | 1 OR 2 |
|  | 4 | 'intensive care nursing'/exp |
|  | 5 | 'critical care nursing':ab,ti OR 'intensive care nursing':ab,ti OR 'ICU nursing':ab,ti OR 'critical care nurse':ab,ti OR 'intensive care nurse':ab,ti OR 'ICU nurse':ab,ti |
|  | 6 | 4 OR 5 |
|  | 7 | 'intensive care'/exp |
|  | 8 | 'critical care':ab,ti OR 'intensive care':ab,ti OR 'critical care medicine':ab,ti OR 'intensive care medicine':ab,ti OR 'critical care unit':ab,ti OR 'intensive care unit':ab,ti OR 'ICU':ab,ti OR 'critical care management':ab,ti |
|  | 9 | 7 OR 8 |
|  | 10 | 3 OR 6 OR 9 |
|  | 11 | 'glycated hemoglobin'/exp |
|  | 12 | 'glycated hemoglobin':ab,ti OR 'glycosylated hemoglobin':ab,ti OR 'HbA1c':ab,ti OR 'A1c':ab,ti OR 'glycemic':ab,ti OR 'glycaemic':ab,ti OR 'glycemia':ab,ti OR 'glycaemia':ab,ti |
|  | 13 | 11 OR 12 |
|  | 14 | 'glucose blood level'/exp |
|  | 15 | 'blood glucose':ab,ti OR 'plasma glucose':ab,ti OR 'blood sugar':ab,ti OR 'plasma sugar':ab,ti OR 'FPG':ab,ti OR 'PPG':ab,ti |
|  | 16 | 14 OR 15 |
|  | 17 | 13 OR 16 |
|  | 18 | 'variability':ab,ti OR 'variabilities':ab,ti OR 'variation':ab,ti OR 'variations':ab,ti OR 'fluctuation':ab,ti OR 'fluctuations':ab,ti OR 'oscillation':ab,ti OR 'oscillations':ab,ti OR 'excursion':ab,ti OR 'excursions':ab,ti |
|  | 19 | 17 AND 18 |
|  | 20 | 'prognosis'/exp |
|  | 21 | 'prognosis':ab,ti OR 'prognostic':ab,ti OR 'prognostication':ab,ti OR 'outcome prediction':ab,ti OR 'clinical prognosis':ab,ti OR 'disease prognosis':ab,ti OR 'predictive outcome':ab,ti |
|  | 22 | 20 OR 21 |
|  | 23 | 'mortality'/exp |
|  | 24 | 'mortality':ab,ti OR 'death rate':ab,ti OR 'death rates':ab,ti OR 'mortality rate':ab,ti OR 'mortality rates':ab,ti OR 'fatality':ab,ti OR 'fatality rate':ab,ti OR 'death ratio':ab,ti |
|  | 25 | 23 OR 24 |
|  | 26 | 'duration of therapy':ab,ti OR 'length of stay':ab,ti OR 'survival rate':ab,ti OR 'therapy duration':ab,ti OR 'duration of treatment':ab,ti OR 'treatment duration':ab,ti OR 'stay length':ab,ti OR 'survival':ab,ti OR 'incidence':ab,ti OR 'outcomes':ab,ti |
|  | 27 | 22 OR 25 OR 26 |
|  | 28 | 10 AND 19 AND 27 |
| **Web of Science** | #1 | TS=("critical illness" OR "severe illness" OR "life-threatening disease" OR "life-threatening condition" OR "critically ill patients" OR "ICU patients" OR "intensive care unit patients") |
|  | #2 | TS=("critical care nursing" OR "intensive care nursing" OR "ICU nursing" OR "critical care nurse" OR "intensive care nurse" OR "ICU nurse") |
|  | #3 | TS=("critical care" OR "intensive care" OR "critical care medicine" OR "intensive care medicine" OR "critical care unit" OR "intensive care unit" OR "ICU" OR "critical care management") |
|  | #4 | #1 OR #2 OR #3 |
|  | #5 | TS=("glycated hemoglobin" OR "glycosylated hemoglobin" OR "HbA1c" OR "A1c" OR "glycemic" OR "glycaemic" OR "glycemia" OR "glycaemia") |
|  | #6 | TS=("blood glucose" OR "plasma glucose" OR "blood sugar" OR "plasma sugar" OR "FPG" OR "PPG") |
|  | #7 | #5 OR #6 |
|  | #8 | TS=("variability" OR "variabilities" OR "variation" OR "variations" OR "fluctuation" OR "fluctuations" OR "oscillation" OR "oscillations" OR "excursion" OR "excursions") |
|  | #9 | #7 AND #8 |
|  | #10 | TS=("prognosis" OR "prognostic" OR "prognostication" OR "outcome prediction" OR "clinical prognosis" OR "disease prognosis" OR "predictive outcome") |
|  | #11 | TS=("mortality" OR "death rate" OR "death rates" OR "mortality rate" OR "mortality rates" OR "fatality" OR "fatality rate" OR "death ratio") |
|  | #12 | TS=("duration of therapy" OR "length of stay" OR "survival rate" OR "therapy duration" OR "duration of treatment" OR "treatment duration" OR "stay length" OR "survival" OR "incidence" OR "outcomes") |
|  | #13 | #10 OR #11 OR #12 |
|  | #14 | #4 AND #9 AND #13 |

^§^ All searches were conducted on 16 August 2025

# **Supplementary Table 3.** Summary of GV metrics.

| **Abbreviation** | **Full name** | **Calculation** |
| --- | --- | --- |
| *Traditional metrics* |  |  |
| SD | Standard Deviation | SD = the square root of the mean squared deviation from the mean glucose level |
| CV | Coefficient of Variation | CV (%) = (SD / mean glucose) × 100% |
| MAG | Mean Absolute Glucose | MAG = the sum of absolute glucose changes divided by the monitoring time (hours) |
| MAGE | Mean Amplitude of Glycemic Excursions | MAGE = the average amplitude of glucose excursions exceeding 1 SD |
| GLI | Glycemic Lability Index | GLI = calculated from weighted squared rates of glucose change |
| J-index | Jensen Index | J-index = 0.324 × (mean glucose + SD)^2^ |
| MODD | Mean of Daily Differences | MODD = mean of absolute day-to-day glucose differences measured at the same time |
| TIR | Time in Range | TIR (%) = (time within the target range / total monitored time) × 100% |
| MAGC | Mean Absolute Glucose Change per hour | MAGC = sum of absolute glucose changes divided by the number of monitoring hours |
| *New metrics* |  |  |
| DGV | Daily Glycemic Variability | DGV = the median of all absolute differences in fasting plasma glucose between consecutive days |
| ACACP | Average Consecutive Absolute Change Percentage | The mean of absolute percentage changes between consecutive glucose values |
| MCACP | Median Consecutive Absolute Change Percentage | The median of absolute percentage changes between consecutive glucose values |

# **Supplementary Methods：**Effect Estimate Standardization

# For each eligible study, we extracted the reported effect measure (HR, RR, or OR), point estimate, 95% CI, GV definition, outcome definition, and exposure contrast (per-unit increase, per-1 SD increase, or categorical comparisons including extreme dichotomies, tertiles, quartiles, and quintiles). The subsequent standardization procedure consisted of two steps.

# Step 1: Standardization of exposure contrasts

# All reported effect estimates were firstly rescaled, whenever feasible, to a common comparison of the highest versus the lowest quartiles of GV (Q4 vs Q1). The standardized log-effect estimate was calculated as:

#

$$\ln(\theta_{Q4vsQ1})=\ln(\theta_{reported})\times k$$

# Where$\theta_{\mathrm{reported}}$ denotes the originally reported effect estimate under the original exposure contrast and may represent an HR, RR, or OR. The conversion factor $k$ was assigned according to the original exposure contrast: 2.54 for per 1-SD increase, 2.54 × SD for per-unit increase, 2.54/1.59 for extreme dichotomies, 2.54/2.18 for tertiles, and 2.54/2.80 for quintiles, where SD denotes the reported or estimated standard deviation of the corresponding GV metric. The standardized effect estimate was then obtained by exponential back-transformation:

$$\theta_{Q4vsQ1}=\exp\left[ \ln(\theta_{reported})\times k \right]$$

The same transformation was applied to the lower and upper bounds of the 95% CI on the log scale.

Step 2: Harmonization of effect measures

After exposure contrast standardization, all effect estimates were harmonized to the RR scale.

Then HR were treated as approximations of RR:

$$RR\approx HR$$

OR were converted to RR using the following formula:

$$RR=\frac{OR}{(1-P_{0})+(P_{0}\times OR)}$$

Where $P_{0}$ represents the outcome incidence in the reference group. When unavailable, the overall outcome incidence was used as a proxy. Studies originally reporting RR required no further transformation. When conversion to a unified RR comparing Q4 versus Q1 was not feasible, the original estimates were retained and synthesized separately.

# **Supplementary Table 4.** List of excluded articles after full-text review.

| **ID** | **Reason** | **Study** |
| --- | --- | --- |
| 1 | No critically ill patients or data on critically ill patients | Ali NA, O'Brien JM Jr, Dungan K, et al. Glucose variability and mortality in patients with sepsis. Crit Care Med. 2008;36(8):2316-2321. doi:10.1097/CCM.0b013e3181810378 |
| 2 | Duplicate dataset | Badawi O, Waite MD, Fuhrman SA, Zuckerman IH. Association between intensive care unit-acquired dysglycemia and in-hospital mortality. Crit Care Med. 2012;40(12):3180-3188. doi:10.1097/CCM.0b013e3182656ae5 |
| 3 | Unrelated to the exposure | Bagshaw SM, Bellomo R, Jacka MJ, et al. The impact of early hypoglycemia and blood glucose variability on outcome in critical illness. Crit Care. 2009;13(3):R91. doi:10.1186/cc7921 |
| 4 | Unrelated to the exposure | Bang HJ, Youn CS, Park KN, et al. Glucose control and outcomes in diabetic and nondiabetic patients treated with targeted temperature management after cardiac arrest. PLoS One. 2024;19(2):e0298632. Published 2024 Feb 8. doi:10.1371/journal.pone.0298632 |
| 5 | Unrelated to the exposure | Bellaver P, Schaeffer AF, Dullius DP, Viana MV, Leitão CB, Rech TH. Association of multiple glycemic parameters at intensive care unit admission with mortality and clinical outcomes in critically ill patients. Sci Rep. 2019;9(1):18498. Published 2019 Dec 6. doi:10.1038/s41598-019-55080-3 |
| 6 | Study design not eligible | Brunner R, Adelsmayr G, Herkner H, Madl C, Holzinger U. Glycemic variability and glucose complexity in critically ill patients: a retrospective analysis of continuous glucose monitoring data. Crit Care. 2012;16(5):R175. Published 2012 Oct 2. doi:10.1186/cc11657 |
| 7 | Duplicate dataset | Cai W, Li Y, Guo K, Wu X, Chen C, Lin X. Association of glycemic variability with death and severe consciousness disturbance among critically ill patients with cerebrovascular disease: analysis of the MIMIC-IV database. Cardiovasc Diabetol. 2023;22(1):315. Published 2023 Nov 16. doi:10.1186/s12933-023-02048-3 |
| 8 | Unrelated to the exposure | Cao H, Gui L, Hu Y, Yang J, Hua P, Yang S. Association between hemoglobin glycation index and adverse outcomes in critically ill patients with myocardial infarction: A retrospective cohort study. Nutr Metab Cardiovasc Dis. 2025;35(6):103973. doi:10.1016/j.numecd.2025.103973 |
| 9 | No critically ill patients or data on critically ill patients | Chao HY, Liu PH, Lin SC, et al. Association of In-Hospital Mortality and Dysglycemia in Septic Patients. PLoS One. 2017;12(1):e0170408. Published 2017 Jan 20. doi:10.1371/journal.pone.0170408 |
| 10 | Duplicate dataset | Chen J, Huang W, Liang N. Blood glucose fluctuation and in-hospital mortality among patients with acute myocardial infarction: eICU collaborative research database. PLoS One. 2024;19(4):e0300323. Published 2024 Apr 26. doi:10.1371/journal.pone.0300323 |
| 11 | Duplicate dataset | Chen Y, Yang Z, Liu Y, et al. Prognostic value of glycaemic variability for mortality in critically ill atrial fibrillation patients and mortality prediction model using machine learning. Cardiovasc Diabetol. 2024;23(1):426. Published 2024 Nov 26. doi:10.1186/s12933-024-02521-7 |
| 12 | Unrelated to the exposure | Chi A, Lissauer ME, Kirchoffner J, Scalea TM, Johnson SB. Effect of glycemic state on hospital mortality in critically ill surgical patients. Am Surg. 2011;77(11):1483-1489. doi:10.1177/000313481107701138 |
| 13 | Without effect estimates | Cooksley, Tim, Thomas McAvoy, and Philip Haji-Michael. "Glucose control in critical care oncology." Journal of the Intensive Care Society 13.4 (2012): 289-292. |
| 14 | Study design not eligible | Coronel-Castañeda, Luz Gardenia, et al. "301: GLYCEMIC VARIABILITY WITHIN 24 HOURS OF ICU ADMISSION AS A PREDICTOR OF ICU MORTALITY: COHORT STUDY." Critical Care Medicine 53.1 (2025). |
| 15 | Unrelated to the exposure | Cueni-Villoz N, Devigili A, Delodder F, et al. Increased blood glucose variability during therapeutic hypothermia and outcome after cardiac arrest. Crit Care Med. 2011;39(10):2225-2231. doi:10.1097/CCM.0b013e31822572c9 |
| 16 | Unrelated to the exposure | Deininger MM, Weiss M, Wied S, et al. Value of Glycemic Indices for Delayed Cerebral Ischemia after Aneurysmal Subarachnoid Hemorrhage: A Retrospective Single-Center Study. Brain Sci. 2024;14(9):849. Published 2024 Aug 23. doi:10.3390/brainsci14090849 |
| 17 | No critically ill patients or data on critically ill patients | Deng J, Li L, Cao F, et al. Systemic Glycemic Variation Predicts Mortality of Acute Ischemic Stroke After Mechanical Thrombectomy: A Prospective Study Using Continuous Glucose Monitoring. Front Neurol. 2022;13:817033. Published 2022 Mar 18. doi:10.3389/fneur.2022.817033 |
| 18 | Without effect estimates | Dossett LA, Cao H, Mowery NT, Dortch MJ, Morris JM Jr, May AK. Blood glucose variability is associated with mortality in the surgical intensive care unit. Am Surg. 2008;74(8):679-685. doi:10.1177/000313480807400802 |
| 19 | Without effect estimates | Farhy LS, Ortiz EA, Kovatchev BP, Mora AG, Wolf SE, Wade CE. Average daily risk range as a measure of glycemic risk is associated with mortality in the intensive care unit: a retrospective study in a burn intensive care unit. J Diabetes Sci Technol. 2011;5(5):1087-1098. Published 2011 Sep 1. doi:10.1177/193229681100500509 |
| 20 | Duplicate dataset | Gao M, Zhong Z, Yue Y, Liu F. Correlation between glycaemic variability and prognosis in diabetic patients with CKD. Endokrynol Pol. 2022;73(6):947-953. doi:10.5603/EP.a2022.0092 |
| 21 | Duplicate dataset | Ge Y, Wang G, Huang Y, Zhang Y. Association between the postoperative glycemic variability and mortality after craniotomy: a retrospective cohort study and development of a mortality prediction model. Front Endocrinol (Lausanne). 2025;16:1613662. Published 2025 Jul 17. doi:10.3389/fendo.2025.1613662 |
| 22 | Duplicate dataset | Guo Y, Qiu Y, Xue T, et al. Association between glycemic variability and short-term mortality in patients with acute kidney injury: a retrospective cohort study of the MIMIC-IV database. Sci Rep. 2024;14(1):5945. Published 2024 Mar 11. doi:10.1038/s41598-024-56564-7 |
| 23 | Unrelated to the exposure | Haehn N, Huehn M, Ralser M, et al. Impact of dysglycemia during the ebb and flow phases of critically ill burn patients: An observational study. Burns. 2025;51(4):107454. doi:10.1016/j.burns.2025.107454 |
| 24 | Duplicate dataset | He HM, Zheng SW, Xie YY, et al. Simultaneous assessment of stress hyperglycemia ratio and glycemic variability to predict mortality in patients with coronary artery disease: a retrospective cohort study from the MIMIC-IV database. Cardiovasc Diabetol. 2024;23(1):61. Published 2024 Feb 9. doi:10.1186/s12933-024-02146-w |
| 25 | Duplicate dataset | Hou Y, Guo X, Yu J. Association between glycemic variability and all-cause mortality in critically ill patients with non-traumatic subarachnoid hemorrhage: a retrospective study based on the MIMIC-IV database. Eur J Med Res. 2025;30(1):235. Published 2025 Apr 4. doi:10.1186/s40001-025-02468-9 |
| 26 | Study design not eligible | Hryciw BN, Ghossein J, Rochwerg B, et al. Glycemic Variability As a Prognostic Factor for Mortality in Patients With Critical Illness: A Systematic Review and Meta-Analysis. Crit Care Explor. 2024;6(1):e1025. Published 2024 Jan 11. doi:10.1097/CCE.0000000000001025 |
| 27 | Duplicate dataset | Hua Y, Chen Z, Cheng L, et al. Association between glycemic variability and acute kidney injury incidence in patients with cerebral infarction: an analysis of the MIMIC-IV database. Front Endocrinol (Lausanne). 2025;16:1615051. Published 2025 Jun 12. doi:10.3389/fendo.2025.1615051 |
| 28 | Without effect estimates | Issarawattana T, Bhurayanontachai R. Maximal Glycemic Difference, the Possible Strongest Glycemic Variability Parameter to Predict Mortality in ICU Patients. Crit Care Res Pract. 2020;2020:5071509. Published 2020 Aug 24. doi:10.1155/2020/5071509 |
| 29 | Unrelated to the exposure | Jacka MJ, Torok-Both CJ, Bagshaw SM. Blood glucose control among critically ill patients with brain injury. Can J Neurol Sci. 2009;36(4):436-442. doi:10.1017/s0317167100007757 |
| 30 | Unrelated to the exposure | Jia N, Liu XX. Impact of hemoglobin glycation index on prognosis in critical patients with acute ischemic stroke: A retrospective cohort study using MIMIC-IV 2.2 database. Sci Rep. 2025;15(1):23095. Published 2025 Jul 2. doi:10.1038/s41598-025-07833-6 |
| 31 | Duplicate dataset | Krinsley JS, Egi M, Kiss A, et al. Diabetic status and the relation of the three domains of glycemic control to mortality in critically ill patients: an international multicenter cohort study. Crit Care. 2013;17(2):R37. Published 2013 Mar 1. doi:10.1186/cc12547 |
| 32 | Duplicate dataset | Krinsley JS, Maurer P, Holewinski S, et al. Glucose Control, Diabetes Status, and Mortality in Critically Ill Patients: The Continuum From Intensive Care Unit Admission to Hospital Discharge. Mayo Clin Proc. 2017;92(7):1019-1029. doi:10.1016/j.mayocp.2017.04.015 |
| 33 | Duplicate dataset | Krinsley JS, Preiser JC. Time in blood glucose range 70 to 140 mg/dl >80% is strongly associated with increased survival in non-diabetic critically ill adults. Crit Care. 2015;19(1):179. Published 2015 Apr 20. doi:10.1186/s13054-015-0908-7 |
| 34 | Duplicate dataset | Krinsley JS. Glycemic variability and mortality in critically ill patients: the impact of diabetes. J Diabetes Sci Technol. 2009;3(6):1292-1301. Published 2009 Nov 1. doi:10.1177/193229680900300609 |
| 35 | Duplicate dataset | Krinsley JS. Glycemic variability: a strong independent predictor of mortality in critically ill patients. Crit Care Med. 2008;36(11):3008-3013. doi:10.1097/CCM.0b013e31818b38d2 |
| 36 | Unrelated to the exposure | Kulkarni H, Bihari S, Prakash S, et al. Independent Association of Glucose Variability With Hospital Mortality in Adult Intensive Care Patients: Results From the Australia and New Zealand Intensive Care Society Centre for Outcome and Resource Evaluation Binational Registry. Crit Care Explor. 2019;1(8):e0025. Published 2019 Aug 1. doi:10.1097/CCE.0000000000000025 |
| 37 | Without effect estimates | Lazzeri C, Bonizzoli M, Batacchi S, Di Valvasone S, Chiostri M, Peris A. The prognostic role of hyperglycemia and glucose variability in covid-related acute respiratory distress Syndrome. Diabetes Res Clin Pract. 2021;175:108789. doi:10.1016/j.diabres.2021.108789 |
| 38 | Duplicate dataset | Lazzeri C, Valente S, Chiostri M, Attanà P, Gensini GF. Early glucose variability in cardiogenic shock following acute myocardial infarction: a pilot study. Ther Adv Cardiovasc Dis. 2015;9(4):127-132. doi:10.1177/1753944715578968 |
| 39 | No critically ill patients or data on critically ill patients | Leung CH, Liu CP. Diabetic status and the relationship of blood glucose to mortality in adults with carbapenem-resistant Acinetobacter baumannii complex bacteremia. J Microbiol Immunol Infect. 2019;52(4):654-662. doi:10.1016/j.jmii.2018.06.005 |
| 40 | Study design not eligible | Ley SC, Kindgen-Milles D. Schwankungen der Blutzuckerkonzentration und Kurzzeit-Letalität bei kritisch kranken Patienten [Variability of blood glucose concentration and short-term mortality in critically ill patient]. Anaesthesist. 2007;56(8):820-821. doi:10.1007/s00101-007-1229-2 |
| 41 | Duplicate dataset | Lin L, Liang Z. Association Between Glycemic Variability and All-Cause Mortality in Patients with Acute Pancreatitis in the Intensive Care Unit: A Retrospective Analysis. Dig Dis Sci. 2025;70(6):2194-2203. doi:10.1007/s10620-025-09012-z |
| 42 | No critically ill patients or data on critically ill patients | Lipska KJ, Venkitachalam L, Gosch K, et al. Glucose variability and mortality in patients hospitalized with acute myocardial infarction. Circ Cardiovasc Qual Outcomes. 2012;5(4):550-557. doi:10.1161/CIRCOUTCOMES.111.963298 |
| 43 | Study design not eligible | Liu WY, Lin SG, Zhu GQ, et al. Establishment and Validation of GV-SAPS II Scoring System for Non-Diabetic Critically Ill Patients. PLoS One. 2016;11(11):e0166085. Published 2016 Nov 8. doi:10.1371/journal.pone.0166085 |
| 44 | Unrelated to the exposure | Liu X, Zhang G, Li D, Ruan Z, Wu B. Effect of 24 h glucose fluctuations on 30-day and 1-year mortality in patients with acute myocardial infarction: an analysis from the MIMIC-III database. Front Cardiovasc Med. 2024;11:1371606. Published 2024 Mar 20. doi:10.3389/fcvm.2024.1371606 |
| 45 | Duplicate dataset | Liu Y, Fu H, Wang Y, et al. U-shaped association between the glycemic variability and prognosis in hemorrhagic stroke patients: a retrospective cohort study from the MIMIC-IV database. Front Endocrinol (Lausanne). 2025;16:1546164. Published 2025 Apr 3. doi:10.3389/fendo.2025.1546164 |
| 46 | Unrelated to the exposure | Lou R, Jiang L, Wang M, et al. The Value of Glycemic Gap for Predicting Mortality in ICU in Patients With and Without Diabetes. J Diabetes Res. 2025;2025:4563928. Published 2025 Feb 14. doi:10.1155/jdr/4563928 |
| 47 | Without effect estimates | Lou R, Jiang L, Zhu B. Effect of glycemic gap upon mortality in critically ill patients with diabetes. J Diabetes Investig. 2021;12(12):2212-2220. doi:10.1111/jdi.13606 |
| 48 | Duplicate dataset | Lu Y, Zhang Q, Lou J. Blood glucose-related indicators are associated with in-hospital mortality in critically ill patients with acute pancreatitis. Sci Rep. 2021;11(1):15351. Published 2021 Jul 28. doi:10.1038/s41598-021-94697-1 |
| 49 | Duplicate dataset | Lu Z, Tao G, Sun X, et al. Association of Blood Glucose Level and Glycemic Variability With Mortality in Sepsis Patients During ICU Hospitalization. Front Public Health. 2022;10:857368. Published 2022 Apr 29. doi:10.3389/fpubh.2022.857368 |
| 50 | Without effect estimates | Lundelin K, Vigil L, Bua S, Gomez-Mestre I, Honrubia T, Varela M. Differences in complexity of glycemic profile in survivors and nonsurvivors in an intensive care unit: a pilot study. Crit Care Med. 2010;38(3):849-854. doi:10.1097/CCM.0b013e3181ce49cf |
| 51 | Unrelated to the exposure | Magee F, Bailey M, Pilcher DV, Mårtensson J, Bellomo R. Early glycemia and mortality in critically ill septic patients: Interaction with insulin-treated diabetes. J Crit Care. 2018;45:170-177. doi:10.1016/j.jcrc.2018.03.012 |
| 52 | Unrelated to the exposure | Mamtani M, Kulkarni H, Bihari S, et al. Degree of hyperglycemia independently associates with hospital mortality and length of stay in critically ill, nondiabetic patients: Results from the ANZICS CORE binational registry. J Crit Care. 2020;55:149-156. doi:10.1016/j.jcrc.2019.11.003 |
| 53 | No critically ill patients or data on critically ill patients | Mellbin LG, Malmberg K, Rydén L, Wedel H, Vestberg D, Lind M. The relationship between glycaemic variability and cardiovascular complications in patients with acute myocardial infarction and type 2 diabetes: a report from the DIGAMI 2 trial. Eur Heart J. 2013;34(5):374-379. doi:10.1093/eurheartj/ehs384 |
| 54 | Study design not eligible | Meyfroidt G, Keenan DM, Wang X, Wouters PJ, Veldhuis JD, Van den Berghe G. Dynamic characteristics of blood glucose time series during the course of critical illness: effects of intensive insulin therapy and relative association with mortality. Crit Care Med. 2010;38(4):1021-1029. doi:10.1097/CCM.0b013e3181cf710e |
| 55 | Without effect estimates | Meynaar IA, Eslami S, Abu-Hanna A, van der Voort P, de Lange DW, de Keizer N. Blood glucose amplitude variability as predictor for mortality in surgical and medical intensive care unit patients: a multicenter cohort study. J Crit Care. 2012;27(2):119-124. doi:10.1016/j.jcrc.2011.11.004 |
| 56 | No critically ill patients or data on critically ill patients | Mi SH, Su G, Yang HX, et al. Comparison of in-hospital glycemic variability and admission blood glucose in predicting short-term outcomes in non-diabetes patients with ST elevation myocardial infarction underwent percutaneous coronary intervention. Diabetol Metab Syndr. 2017;9:20. Published 2017 Mar 21. doi:10.1186/s13098-017-0217-1 |
| 57 | No critically ill patients or data on critically ill patients | Miyoshi M, Uzui H, Shimizu T, et al. Significance of day-to-day glucose variability in patients after acute coronary syndrome. BMC Cardiovasc Disord. 2021;21(1):490. Published 2021 Oct 10. doi:10.1186/s12872-021-02303-z |
| 58 | Unrelated to the exposure | Oh TK, Heo E, Song IA, Jeong WJ, Han M, Bang JS. Increased Glucose Variability During Long-Term Therapeutic Hypothermia as a Predictor of Poor Neurological Outcomes and Mortality: A Retrospective Study. Ther Hypothermia Temp Manag. 2020;10(2):106-113. doi:10.1089/ther.2019.0004 |
| 59 | Without effect estimates | Oliveira AP, Castro MDS, Lima DVM. Glycemic variability and mortality in oncologic intensive care units. Rev Bras Enferm. 2023;76(4):e20220812. Published 2023 Oct 9. doi:10.1590/0034-7167-2022-0812 |
| 60 | No critically ill patients or data on critically ill patients | Palaiodimou L, Lioutas VA, Lambadiari V, et al. Glycemic variability of acute stroke patients and clinical outcomes: a continuous glucose monitoring study. Ther Adv Neurol Disord. 2021;14:17562864211045876. Published 2021 Sep 22. doi:10.1177/17562864211045876 |
| 61 | Unrelated to the exposure | Pan L, Lu F, Cheng B, Zhang W, Wang B. Association between hemoglobin glycation index and mortality in critically ill patients: a retrospective cohort study. J Health Popul Nutr. 2025;44(1):249. Published 2025 Jul 12. doi:10.1186/s41043-025-01008-9 |
| 62 | Unrelated to the exposure | Pappacena S, Bailey M, Cabrini L, et al. Early dysglycemia and mortality in traumatic brain injury and subarachnoid hemorrhage. Minerva Anestesiol. 2019;85(8):830-839. doi:10.23736/S0375-9393.19.13307-X |
| 63 | Duplicate dataset | Peng J, Zhang X, Mai Y, et al. A retrospective analysis of the prognostic implications of glycemic variability on all-cause mortality in critically ill patients with mitral valve disease. Front Endocrinol (Lausanne). 2025;16:1620762. Published 2025 Jul 14. doi:10.3389/fendo.2025.1620762 |
| 64 | Unrelated to the exposure | Penning S, Pretty C, Preiser JC, Shaw GM, Desaive T, Chase JG. Glucose control positively influences patient outcome: A retrospective study. J Crit Care. 2015;30(3):455-459. doi:10.1016/j.jcrc.2014.12.013 |
| 65 | Unrelated to the exposure | Pidcoke HF, Wanek SM, Rohleder LS, Holcomb JB, Wolf SE, Wade CE. Glucose variability is associated with high mortality after severe burn. J Trauma. 2009;67(5):990-995. doi:10.1097/TA.0b013e3181baef4b |
| 66 | Without effect estimates | Plummer MP, Finnis ME, Horsfall M, et al. Prior exposure to hyperglycaemia attenuates the relationship between glycaemic variability during critical illness and mortality. Crit Care Resusc. 2016;18(3):189-197. |
| 67 | Duplicate dataset | Qi L, Geng X, Feng R, et al. Association of glycemic variability and prognosis in patients with traumatic brain injury: A retrospective study from the MIMIC-IV database. Diabetes Res Clin Pract. 2024;217:111869. doi:10.1016/j.diabres.2024.111869 |
| 68 | Study design not eligible | Rodbard D. Glucose Variability: A Review of Clinical Applications and Research Developments. Diabetes Technol Ther. 2018;20(S2):S25-S215. doi:10.1089/dia.2018.0092 |
| 69 | Study design not eligible | Sadan, O., Hall, C.L. (2023). Glucose Variability Measures in Critical Care. In: Rajendram, R., Preedy, V.R., Patel, V.B. (eds) Biomarkers in Trauma, Injury and Critical Care. Biomarkers in Disease: Methods, Discoveries and Applications. Springer, Cham. https://doi.org/10.1007/978-3-031-07395-3_24 |
| 70 | Study design not eligible | Service FJ. Glucose variability. Diabetes. 2013;62(5):1398-1404. doi:10.2337/db12-1396 |
| 71 | No critically ill patients or data on critically ill patients | Shen Y, Fan X, Zhang L, et al. Thresholds of Glycemia and the Outcomes of COVID-19 Complicated With Diabetes: A Retrospective Exploratory Study Using Continuous Glucose Monitoring. Diabetes Care. 2021;44(4):976-982. doi:10.2337/dc20-1448 |
| 72 | Duplicate dataset | Shuai WL, Zhang HJ, Wang N, et al. Association of glycemic variability with short and long-term mortality among critically ill patients with heart failure: Analysis of the MIMIC-IV database. Diabetes Res Clin Pract. 2025;221:112009. doi:10.1016/j.diabres.2025.112009 |
| 73 | Without effect estimates | Singh M, Upreti V, Singh Y, Kannapur AS, Nakra M, Kotwal N. Effect of Glycemic Variability on Mortality in ICU Settings: A Prospective Observational Study. Indian J Endocrinol Metab. 2018;22(5):632-635. doi:10.4103/ijem.IJEM_11_18 |
| 74 | Duplicate dataset | Tang S, Zhang Z, Cheng Y, Zhang L, Wang Q, Wang C. Glycemic variability and mortality in patients with aortic diseases: A multicenter retrospective cohort study. PLoS One. 2025;20(6):e0325006. Published 2025 Jun 25. doi:10.1371/journal.pone.0325006 |
| 75 | Without effect estimates | Uijtendaal EV, Zwart-van Rijkom JE, de Lange DW, Lalmohamed A, van Solinge WW, Egberts TC. Influence of a strict glucose protocol on serum potassium and glucose concentrations and their association with mortality in intensive care patients. Crit Care. 2015;19(1):270. Published 2015 Jun 22. doi:10.1186/s13054-015-0959-9 |
| 76 | Unrelated to the exposure | Uyttendaele V, Dickson JL, Shaw GM, Desaive T, Chase JG. Untangling glycaemia and mortality in critical care. Crit Care. 2017;21(1):152. Published 2017 Jun 24. doi:10.1186/s13054-017-1725-y |
| 77 | Without effect estimates | van Keulen K, Knol W, Belitser SV, et al. Glucose variability during delirium in diabetic and non-diabetic intensive care unit patients: A prospective cohort study. PLoS One. 2018;13(11):e0205637. Published 2018 Nov 15. doi:10.1371/journal.pone.0205637 |
| 78 | No critically ill patients or data on critically ill patients | Wada S, Yoshimura S, Inoue M, et al. Outcome Prediction in Acute Stroke Patients by Continuous Glucose Monitoring. J Am Heart Assoc. 2018;7(8):e008744. Published 2018 Apr 12. doi:10.1161/JAHA.118.008744 |
| 79 | Without effect estimates | Waeschle RM, Moerer O, Hilgers R, Herrmann P, Neumann P, Quintel M. The impact of the severity of sepsis on the risk of hypoglycaemia and glycaemic variability. Crit Care. 2008;12(5):R129. doi:10.1186/cc7097 |
| 80 | Unrelated to the exposure | Wang C, Wang W, Li G, et al. Prognostic value of glycemic gap in patients with spontaneous intracerebral hemorrhage. Eur J Neurol. 2022;29(9):2725-2733. doi:10.1111/ene.15432 |
| 81 | Duplicate dataset | Wang D, He C, Zou S, et al. Glycemic variability and its association with short and long term clinical outcomes in critically ill patients with cerebral hemorrhage. Sci Rep. 2025;15(1):7820. Published 2025 Mar 6. doi:10.1038/s41598-025-92415-9 |
| 82 | Duplicate dataset | Wang F, Guo Y, Tang Y, et al. Combined assessment of stress hyperglycemia ratio and glycemic variability to predict all-cause mortality in critically ill patients with atherosclerotic cardiovascular diseases across different glucose metabolic states: an observational cohort study with machine learning. Cardiovasc Diabetol. 2025;24(1):199. Published 2025 May 9. doi:10.1186/s12933-025-02762-0 |
| 83 | Duplicate dataset | Wang F, Mei X. Association of blood glucose change with postoperative delirium after coronary artery bypass grafting in patients with diabetes mellitus: a study of the MIMIC-IV database. Front Endocrinol (Lausanne). 2024;15:1400207. Published 2024 Jun 20. doi:10.3389/fendo.2024.1400207 |
| 84 | Unrelated to the exposure | Wang Y, Li S, Lu J, et al. The complexity of glucose time series is associated with short- and long-term mortality in critically ill adults: a multi-center, prospective, observational study. J Endocrinol Invest. 2024;47(12):3091-3099. doi:10.1007/s40618-024-02393-4 |
| 85 | Study design not eligible | Xie Y, Lin J, Gallagher M, et al. Prognostic Significance of Baseline Blood Glucose Levels and Glucose Variability in Severe Acute Kidney Injury: A Secondary Analysis from the RENAL Study. J Clin Med. 2022;12(1):15. Published 2022 Dec 20. doi:10.3390/jcm12010015 |
| 86 | Unrelated to the exposure | YADAV, BHARAT, VINOD KUMAR, and AMIT NACHANKAR. "Analysis of Glycaemic Changes and their Outcome in Critically Ill Non-diabetic Patients Admitted to the ICU: A Cohort Study." Journal of Clinical & Diagnostic Research 17.11 (2023). |
| 87 | Duplicate dataset | Yang H, Wang H, Jiang Y. Exploring the impact of glycemic variability on clinical outcomes in critically ill cerebral infarction patients. Diabetol Metab Syndr. 2025;17(1):100. Published 2025 Mar 25. doi:10.1186/s13098-025-01676-x |
| 88 | Duplicate dataset | Yang Z, Li Y, Liu Y, et al. Prognostic effects of glycaemic variability on diastolic heart failure and type 2 diabetes mellitus: insights and 1-year mortality machine learning prediction model. Diabetol Metab Syndr. 2024;16(1):280. Published 2024 Nov 23. doi:10.1186/s13098-024-01534-2 |
| 89 | Unrelated to the exposure | Yavuz, Ozgur Diyar, et al. "The effect of blood glucose variability on the survival of patients in the intensive care unit: a prospective observational study." Signa Vitae 19.4 (2023). |
| 90 | No critically ill patients or data on critically ill patients | You H, Hou X, Zhang H, et al. Effect of glycemic control and glucose fluctuation on in-hospital adverse outcomes after on-pump coronary artery bypass grafting in patients with diabetes: a retrospective study. Diabetol Metab Syndr. 2023;15(1):20. Published 2023 Feb 14. doi:10.1186/s13098-023-00984-4 |
| 91 | Duplicate dataset | Yu Q, Fu Q, Ma X, et al. Impact of glycemic control metrics on short- and long-term mortality in transcatheter aortic valve replacement patients: a retrospective cohort study from the MIMIC-IV database. Cardiovasc Diabetol. 2025;24(1):135. Published 2025 Mar 22. doi:10.1186/s12933-025-02684-x |
| 92 | Duplicate dataset | Zhang X, Zhang J, Li J, et al. Relationship between 24-h venous blood glucose variation and mortality among patients with acute respiratory failure. Sci Rep. 2021;11(1):7747. Published 2021 Apr 8. doi:10.1038/s41598-021-87409-2 |
| 93 | Duplicate dataset | Zhang Z, Ji M, Zhao Q, Jiang L, Fan S, Zuo H. Predictive value of glucose coefficient of variation for in-hospital mortality in acute myocardial infarction patients undergoing PCI: Insights from the MIMIC-IV database. Int J Cardiol Cardiovasc Risk Prev. 2024;23:200347. Published 2024 Oct 26. doi:10.1016/j.ijcrp.2024.200347 |
| 94 | Without effect estimates | Zhou J, Chen Z, Huang HN, Ou CQ, Li X. Association between various blood glucose variability-related indicators during early ICU admission and 28-day mortality in non-diabetic patients with sepsis. Diabetol Metab Syndr. 2025;17(1):22. Published 2025 Jan 20. doi:10.1186/s13098-025-01580-4 |

# **Supplementary Table 5.** Quality scores of the included articles (n = 36).

| **Study, listed by first author** | **Selection**^§^ | | | | **Comparability**^§^ | | **Outcome**^§^ | | | **Total NOS score^§^** |
| --- | --- | --- | --- | --- | --- | --- | --- | --- | --- | --- |
|  | **Sample Representativeness** | **Comparison Selection** | **Exposure Ascertainment** | **Outcome Absence** | **Primary Control** | **Secondary Control** | **Outcome Assessment** | **Follow-up Duration** | **Follow-up Adequacy** |  |
| Ammar | 1 | 1 | 1 | 1 | 1 | 1 | 1 | 1 | 1 | 9 |
| Bansal | 1 | 1 | 1 | 0 | 0 | 1 | 1 | 1 | 0 | 6 |
| Cai | 1 | 1 | 1 | 1 | 0 | 1 | 1 | 1 | 0 | 7 |
| Chao | 1 | 1 | 1 | 1 | 1 | 1 | 1 | 1 | 0 | 8 |
| Dahagam | 1 | 1 | 1 | 1 | 1 | 1 | 1 | 1 | 1 | 9 |
| Donati | 1 | 1 | 1 | 1 | 0 | 1 | 1 | 1 | 1 | 8 |
| Doola | 1 | 1 | 1 | 1 | 1 | 1 | 1 | 1 | 1 | 9 |
| Egi | 1 | 1 | 1 | 1 | 1 | 1 | 1 | 1 | 1 | 9 |
| Emgin | 1 | 1 | 1 | 1 | 1 | 1 | 1 | 1 | 0 | 8 |
| Fong | 1 | 1 | 1 | 1 | 1 | 1 | 1 | 1 | 1 | 9 |
| Furushima | 1 | 1 | 1 | 1 | 1 | 0 | 1 | 1 | 0 | 7 |
| Gerbaud | 1 | 1 | 1 | 1 | 1 | 1 | 1 | 1 | 1 | 9 |
| Gunawan | 1 | 1 | 1 | 1 | 0 | 1 | 1 | 1 | 0 | 7 |
| Hanna | 1 | 1 | 1 | 1 | 1 | 1 | 1 | 1 | 1 | 9 |
| Hartmann | 1 | 1 | 1 | 1 | 1 | 1 | 1 | 1 | 1 | 9 |
| Hermanides | 1 | 1 | 1 | 1 | 1 | 1 | 1 | 1 | 1 | 9 |
| Hoang | 1 | 1 | 1 | 1 | 0 | 1 | 1 | 1 | 1 | 8 |
| Kim | 1 | 1 | 1 | 1 | 1 | 1 | 1 | 1 | 0 | 8 |
| Krinsley | 1 | 1 | 1 | 1 | 0 | 1 | 1 | 1 | 1 | 8 |
| Kurtz | 1 | 1 | 1 | 0 | 1 | 1 | 1 | 1 | 0 | 7 |
| Lanspa | 1 | 1 | 1 | 1 | 1 | 1 | 1 | 1 | 0 | 8 |
| Lazzeri | 1 | 1 | 1 | 1 | 0 | 1 | 1 | 1 | 1 | 8 |
| Lazzeri | 1 | 1 | 1 | 1 | 0 | 1 | 1 | 1 | 1 | 8 |
| Li | 1 | 1 | 1 | 1 | 0 | 1 | 1 | 1 | 0 | 7 |
| Liu | 1 | 1 | 1 | 0 | 1 | 1 | 1 | 1 | 0 | 7 |
| Ma | 1 | 1 | 1 | 1 | 1 | 1 | 1 | 1 | 1 | 9 |
| Okazaki | 1 | 1 | 1 | 1 | 0 | 1 | 1 | 1 | 1 | 8 |
| Okazaki | 1 | 1 | 1 | 1 | 1 | 1 | 1 | 1 | 1 | 9 |
| Réa | 1 | 1 | 1 | 1 | 1 | 1 | 1 | 1 | 0 | 8 |
| Sadan | 1 | 1 | 1 | 1 | 1 | 1 | 1 | 1 | 0 | 8 |
| Sechterberger | 1 | 1 | 1 | 1 | 1 | 1 | 1 | 1 | 1 | 9 |
| Sundarsingh | 1 | 1 | 1 | 1 | 0 | 1 | 1 | 1 | 0 | 7 |
| Todi | 1 | 1 | 1 | 1 | 0 | 1 | 1 | 1 | 1 | 9 |
| Yao | 1 | 1 | 1 | 1 | 0 | 1 | 1 | 1 | 0 | 7 |
| Zhu | 1 | 1 | 1 | 1 | 1 | 1 | 1 | 1 | 0 | 8 |
| Zuo | 1 | 1 | 1 | 1 | 1 | 1 | 1 | 1 | 1 | 9 |

^§^ NOS, Newcastle–Ottawa Scale.

Sample Representativeness: the exposed cohort is truly or somewhat representative of the average community member rather than a highly specialized or "selected" group.

Comparison Selection: the non-exposed cohort is drawn from the same community or source as the exposed cohort.

Exposure Ascertainment: the exposure status is verified through secure records (e.g., medical records) or structured interviews rather than simple self-reports.

Outcome Absence: the study demonstrates the outcome of interest was definitively not present at the start of the study.

Primary Control: the study design or analysis controls for the most important factor, typically age and sex.

Secondary Control: the study also controls for any additional important factor, such as illness severity (e.g., SAPS, APACHE, or ISS scores).

Outcome Assessment: the outcome is assessed through independent blind assessment or record linkage (e.g., ICD codes) rather than unverified self-reports.

Follow-up Duration: the follow-up period was long enough for the outcome of interest to realistically occur in the participants.

Follow-up Adequacy: all subjects are accounted for or if the number lost to follow-up is small enough (typically <10% to 20%) to be unlikely to introduce significant bias.

# **Supplementary Table 6.** Detailed effect estimates of the included articles (n = 36).

| **Author** | **Year** | **Outcome** | **Population/Subgroup** | **GV metric** | **Comparison** | **Effect** | **95% CI lower** | **95% CI upper** | **P** | **Adjusted** |
| --- | --- | --- | --- | --- | --- | --- | --- | --- | --- | --- |
| Ammar | 2022 | Hospital mortality | Non-DM ICU patients | TIR | High vs low | 0.52 | 0.27 | 0.97 | 0.03 | Yes |
| Bansal | 2016 | Prolonged LOS-ICU | All patients | SD | Per unit | 1.016 | 1.01 | 1.03 | 0.006 | Yes |
|  |  | Prolonged LOS-Hospital | All patients | SD | Per unit | 1 | 0.99 | 1.02 | 0.99 | Yes |
|  |  | Infection | All patients | SD | Per unit | 1.02 | 1 | 1.03 | 0.057 | Yes |
|  |  | Readmission | All patients | SD | Per unit | 1.01 | 0.99 | 1.03 | 0.33 | Yes |
|  |  | AKI | All patients | SD | Per unit | 1.034 | 1.02 | 1.05 | <0.001 | Yes |
| Cai | 2020 | 3 month mortality | All patients | SD | Per unit | 1.14 | 1.02 | 1.28 | 0.028 | Yes |
|  |  | 3 month mortality | All patients | MAGE | Per unit | 1.38 | 1.04 | 1.83 | 0.027 | Yes |
|  |  | 3 month mortality | All patients | CV | Per unit | 0.94 | 0.73 | 1.21 | 0.03 | Yes |
| Chao | 2020 | 30-day mortality | All patients | MAGE | High vs Low | 1.607 | 1.008 | 2.563 | 0.04 | Yes |
|  |  | 30-day mortality | All patients | CV | High vs Low | 2.593 | 1.494 | 4.499 | <0.01 | Yes |

| Dahagam | 2011 | ICU-free days | All patients | CV | Per unit | −0.03 | −0.12 | 0.06 | 0.49 | Yes |
| --- | --- | --- | --- | --- | --- | --- | --- | --- | --- | --- |
|  |  | Ventilator-free days | All patients | CV | Per unit | 0.06 | −0.02 | 0.15 | 0.15 | Yes |
|  |  | Hospital-free days | All patients | CV | Per unit | −0.24 | −0.40 | −0.08 | <0.05 | Yes |
| Donati | 2014 | ICU mortality | All patients | GLI | Extreme quartiles | 1.851 | 1.257 | 2.726 | 0.002 | Yes |
|  |  | ICU-acquired infection | All patients | GLI | Extreme quartiles | 2.271 | 1.635 | 3.155 | <0.001 | Yes |
| Doola | 2018 | ICU mortality | All patients | CV | Per unit | 1.02 | 1 | 1.04 | 0.03 | Yes |
| Egi | 2006 | ICU mortality | All patients | SD | Per unit | 1.28 | 1.14 | 1.44 | 0.004 | Yes |
|  |  | Hospital mortality | All patients | SD | Per unit | 1.18 | 1.07 | 1.31 | 0.017 | Yes |
| Emgin | 2024 | 28-day mortality | All patients | CV | Per unit | 1.023 | 1.004 | 1.042 | 0.017 | Yes |
| Fong | 2022 | Hospital mortality | Non-DM ICU patients | CV | Extreme quintiles | 2.5 | 2.37 | 2.65 | <0.001 | Yes |
|  |  | Hospital mortality | DM ICU patients | CV | Extreme quintiles | 1.05 | 1.04 | 1.06 | <0.001 | Yes |
| Furushima | 2021 | 90-day mortality | All patients | MAGE | Per unit | 1.041 | 1.006 | 1.078 | 0.02 | Yes |
|  |  | ICU-free days | All patients | MAGE | Per unit | –0.51 | –0.90 | –0.12 | 0.03 | Yes |
| Gerbaud | 2022 | MACE | All patients | SD | High vs low | 3.16 | 2.25 | 4.43 | <0.001 | Yes |
| Gunawan | 2025 | 30-day mortality | All patients | MAGE | High vs low | 68.49 | 17.27 | 271.25 | <0.001 | No |
| Hanna | 2021 | Hospital mortality | All patients | GLI | High vs low | 1.6 | 1.19 | 2.15 | 0.002 | Yes |
| Hartmann | 2022 | ICU mortality | All patients | DGV | Per unit | 1.02 | 1.007 | 1.03 | <0.001 | Yes |
| Hermanides | 2010 | ICU mortality | All patients | MAG | Extreme quartiles | 3.3 | 2.1 | 5.4 | <0.001 | Yes |
|  |  | Hospital mortality | All patients | MAG | Extreme quartiles | 2.8 | 2 | 3.9 | <0.001 | Yes |
| Hoang | 2024 | Composite complications | All patients | J-index | Per unit | 1.044 | 1.002 | 1.087 | NR | Yes |
|  |  | infection | All patients | J-index | Per unit | 1.049 | 1.007 | 1.093 | NR | Yes |
|  |  | Composite complications | All patients | SD | Per unit | 1.031 | 0.993 | 1.069 | NR | Yes |
|  |  | infection | All patients | SD | Per unit | 1.042 | 1.003 | 1.084 | NR | Yes |
|  |  | Composite complications | All patients | CV | Per unit | 1.041 | 0.976 | 1.11 | NR | Yes |
|  |  | Composite complications | All patients | MAGE | Per unit | 1.009 | 0.99 | 1.028 | NR | Yes |
| Kim | 2022 | 28-day mortality | All patients | CV | Per unit | 1.01 | 1 | 1.02 | 0.04 | Yes |
|  |  | prolonged ICU stay | All patients | CV | Per unit | 1.02 | 1 | 1.04 | 0.04 | Yes |
| Krinsley | 2020 | Hospital mortality | HbA1c < 6.5% | CV | Extreme tertiles | 1.6 | 1.17 | 2.22 | 0.0038 | Yes |
|  |  | Hospital mortality | HbA1c 6.5–7.9% | CV | Extreme tertiles | 0.95 | 0.4 | 2.25 | 0.9 | Yes |
|  |  | Hospital mortality | HbA1c ≥8.0% | CV | Extreme tertiles | 0.48 | 0.22 | 1.05 | 0.067 | Yes |
| Kurtz | 2014 | Hospital mortality | All patients | SD | Per unit | 10.4 | 1.3 | 86 | 0.03 | Yes |
|  |  | CMD | All patients | SD | Per unit | 1.5 | 1.1 | 2.1 | 0.02 | Yes |
| Lanspa | 2014 | 30-day mortality | All patients | CV | Per 10 unit | 1.23 | 1.16 | 1.31 | <0.001 | Yes |
| Lazzeri | 2020 | ICU mortality | All patients | SD | Per unit | 1.069 | 1.013 | 1.129 | 0.016 | Yes |
|  |  | ICU mortality | All patients | CV | Per unit | 1.097 | 1.015 | 1.186 | 0.02 | Yes |
| Lazzeri | 2014 | Follow up mortality | All patients | SD | Per unit | 2.91 | 1.16 | 7.33 | 0.023 | Yes |
|  |  | Follow up mortality | All patients | MAGC | Per unit | 3.94 | 1.34 | 11.56 | 0.012 | Yes |
| Li | 2019 | Arrhythmia | All patients | MODD | High vs low | 1.513 | 1.14 | 2.01 | 0.004 | Yes |
| Liu | 2022 | DIC | All patients | GLI | Per unit | 1.866 | 1.41 | 2.47 | <0.01 | Yes |
| Ma | 2022 | Hospital mortality | Non-diabetic patients | CV | Per unit | 1.05 | 1.02 | 1.08 | <0.001 | Yes |
|  |  | Hospital mortality | Diabetic patients | CV | Per unit | 1.06 | 1.02 | 1.11 | <0.01 | Yes |
| Okazaki | 2016 | Unfavorable neurological outcome | All patients | SD | Per unit | 1.09 | 1.02 | 1.17 | <0.01 | Yes |
| Okazaki | 2022 | Hospital mortality | All patients | TIR OF RN | High vs low | 0.16 | 0.06 | 0.43 | <0.001 | Yes |
|  |  | 28-day mortality | All patients | TIR OF RN | High vs low | 0.21 | 0.08 | 0.58 | 0.002 | Yes |
|  |  | Hospital mortality | All patients | TIR OF AN | High vs low | 0.44 | 0.15 | 1.23 | 0.118 | Yes |
| Réa | 2023 | 30-day mortality | All patients | CV | Per unit | 1.032 | 1.013 | 1.051 | 0.001 | Yes |

| Sadan | 2020 | Hospital mortality | All patients | ACACP | Per unit | 5.18 | 1.374 | 19.821 | <0.05 | Yes |
| --- | --- | --- | --- | --- | --- | --- | --- | --- | --- | --- |
|  |  | Hospital mortality | All patients | MCACP | Per unit | 8.818 | 1.801 | 43.562 | <0.01 | Yes |
| Sechterberger | 2013 | ICU mortality | Non-DM patients | MAG | Extreme quartiles | 1.69 | 1.2 | 2.3 | 0.001 | Yes |
| Sundarsingh | 2023 | 28-day mortality | ICU patients | SD | High vs low | 1.055 | 0.84 | 1.32 | 0.64 | Yes |
|  |  | 28-day mortality | ICU patients | CV | High vs low | 0.986 | 0.88 | 1.1 | 0.81 | Yes |
|  |  | 28-day mortality | ICU patients | GLI | High vs low | 2.99 | 1.04 | 8.6 | 0.042 | Yes |
|  |  | 28-day mortality | ICU patients | TIR | High vs low | 0.718 | 0.311 | 1.657 | 0.437 | Yes |
|  |  | Bloodstream infections | ICU patients | SD | High vs low | 0.58 | 0.25 | 1.35 | 0.21 | No |
|  |  | Bloodstream infections | ICU patients | CV | High vs low | 0.64 | 0.54 | 1.45 | 0.29 | No |
|  |  | Bloodstream infections | ICU patients | GLI | High vs low | 0.84 | 0.37 | 1.92 | 0.67 | No |
|  |  | Bloodstream infections | ICU patients | TIR | High vs low | 0.31 | 0.13 | 0.74 | 0.009 | No |
| Todi | 2014 | ICU mortality | All patients | SD | Extreme quartiles | 2.264 | 1.755 | 2.92 | 0.0001 | No |
|  |  | ICU mortality | All patients Low-MBG | GLI | High vs Low | 5.62 | 3.865 | 8.198 | <0.001 | No |
| Yao | 2023 | PICS | All patients | CV | Per unit | 1.089 | 1.03 | 1.151 | 0.003 | Yes |

| Zhu | 2025 | All cause mortality | All patients | MAGE | Q4 vs Q1 | 2.62 | 2.34 | 2.94 | <0.001 | Yes |
| --- | --- | --- | --- | --- | --- | --- | --- | --- | --- | --- |
|  |  | ICU mortality | All patients | MAGE | Q4 vs Q1 | 3.59 | 2.99 | 4.31 | <0.001 | Yes |
|  |  | Hospital mortality | All patients | MAGE | Q4 vs Q1 | 3.43 | 2.92 | 4.02 | <0.001 | Yes |
|  |  | 28-day mortality | All patients | MAGE | Q4 vs Q1 | 2.04 | 1.47 | 2.82 | <0.001 | Yes |
|  |  | All cause mortality | All patients | MAGE | Per SD increase | 1.28 | 1.25 | 1.32 | <0.001 | Yes |
|  |  | ICU mortality | All patients | MAGE | Per SD increase | 1.32 | 1.27 | 1.37 | <0.001 | Yes |
|  |  | Hospital mortality | All patients | MAGE | Per SD increase | 1.31 | 1.27 | 1.35 | <0.001 | Yes |
|  |  | 28-day mortality | All patients | MAGE | Per SD increase | 1.21 | 1.12 | 1.3 | <0.001 | Yes |
| Zuo | 2012 | ICU mortality | All patients | GLI | Extreme quartiles | 3.47 | 1.76 | 6.86 | <0.001 | No |
|  |  | Hospital mortality | All patients | GLI | Extreme quartiles | 3.57 | 1.81 | 7.06 | <0.001 | No |

# **Supplementary Table 7.** Effect estimates for forest plot: extracted and converted effect estimates from included studies.

| **Study** | **Effect estimate (RR/OR, 95% CI)** |
| --- | --- |
|  | CV with 28/30-day Mortality |
| Chao et al. (2020) | 4.58 (1.9–11.05) |
| Emgin et al. (2024) | 1.35 (1.06–1.66) |
| Kim et al. (2022) | 1.06 (1–1.12) |
| Lanspa et al. (2014) | 1.46 (1.32–1.63) |
| Réa et al. (2018) | 1.75 (1.31–2.09) |
| Sundarsingh et al. (2023) | 0.98 (0.87–1.1) |
|  | MAGE with 28/30-day Mortality |
| Chao et al. (2020) | 2.13 (1.01–4.5) |
| Zhu et al. (2025) | 2.04^†^ (1.47^†^ –2.82^†^ ) |
|  | MAGE with 90-day Mortality |
| Cai et al. (2020) | 2.49 (1.13–4.22) |
| Furushima et al. (2021) | 3.16 (1.34–3.59) |
|  | CV with ICU Mortality |
| Doola et al. (2018) | 1.41 (1–1.93) |
| Lazzeri et al. (2020) | 3.7 (1.31–5.35) |
|  | SD with ICU Mortality |
| Egi et al. (2006) | 2.16 (1.53–2.95) |
| Lazzeri et al. (2020) | 2.85 (1.27–4.51) |
|  | MAG with ICU Mortality |
| Hermanides et al. (2010) | 3.16 (2.06–4.97) |
| Sechterberger et al. (2013) | 1.65 (1.19–2.21) |
|  | GLI with ICU Mortality |
| Todi et al. (2014) (Low-MBG)^§^ | 7.75 (5.64–9.78) |
| Zuo et al. (2012) | 1.93 (1.41–2.37) |
|  | CV with Hospital Mortality |
| Fong et al. (2022) (Non-DM)^§^ | 1.63 (1.58–1.68) |
| Fong et al. (2022) (DM)^§^ | 1.03 (1.02–1.03) |
| Krinsley et al. (2020) (HbA1c < 6.5 %)^§^ | 1.65 (1.19–2.3) |
| Krinsley et al. (2020) (HbA1c 6.5–7.9%)^§^ | 0.95 (0.37–2.21) |
| Krinsley et al. (2020) (HbA1c ≥8.0%)^§^ | 0.34 (0.1–1.07) |
| Ma et al. (2022) (Non-DM)^§^ | 1.7 (1.29–2.03) |
| Ma et al. (2022) (DM)^§^ | 2 (1.33–2.59) |
|  | SD with Hospital Mortality |
| Egi et al. (2006) | 1.7 (1.25–2.3) |
| Kurtz et al. (2014) | 3.3 (1.24–3.92) |
|  | SD with Infection |
| Bansal et al. (2016) | 1.02^¶^ (1^¶^–1.03^¶^) |
| Hoang et al. (2024) | 1.042^¶^ (1.003^¶^–1.084^¶^) |
|  | SD with Neurological Adverse Events |
| Kurtz et al. (2014) | 1.5^¶^ (1.1^¶^–2.1^¶^) |
| Okazaki et al. (2016) | 1.09^¶^ (1.02^¶^–1.17^¶^) |

^†^RR, Extracted effect estimates. ^§^Patient group: MBG, mean blood glucose; DM, diabetes mellitus.

^¶^OR, Extracted effect estimates.


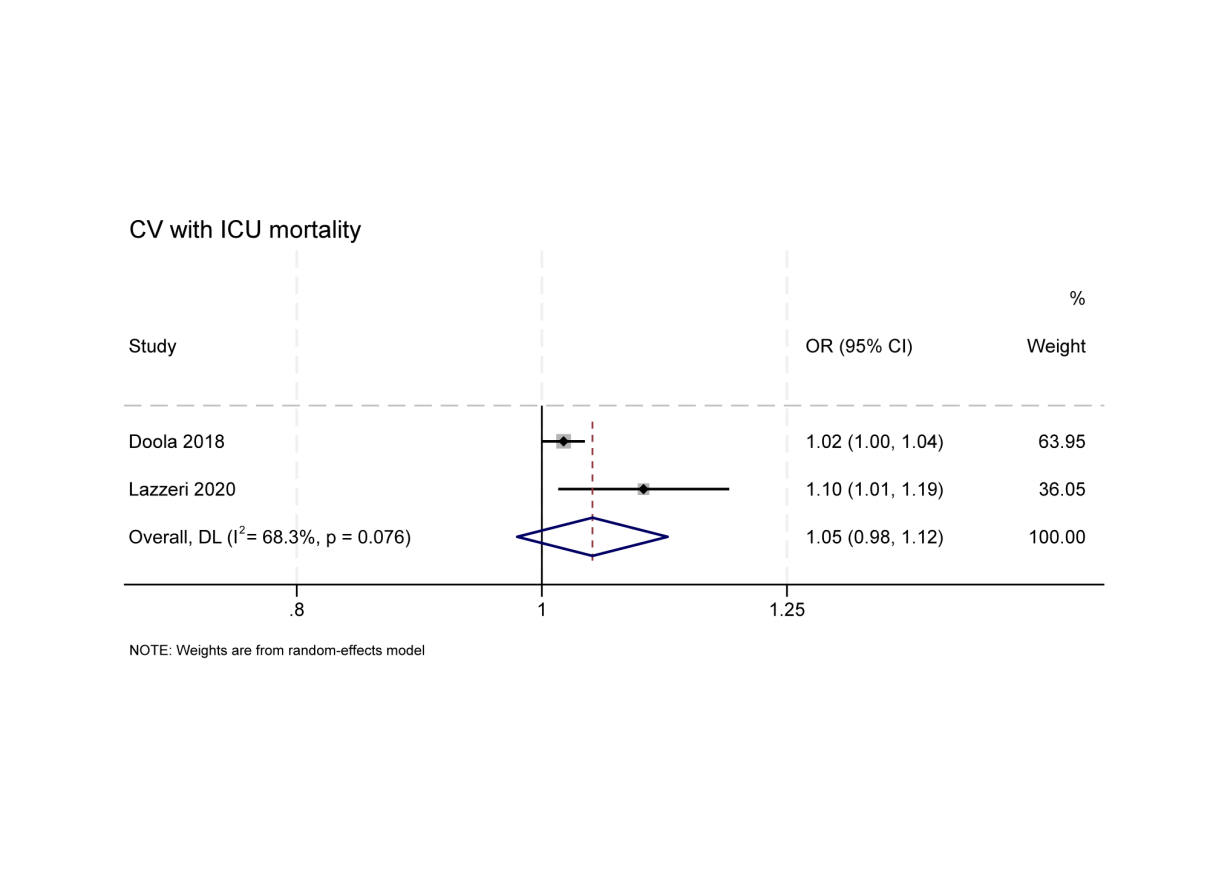


Supplementary Figure 1. Forest plots of CV (per unit increase) and ICU mortality using original OR estimates.


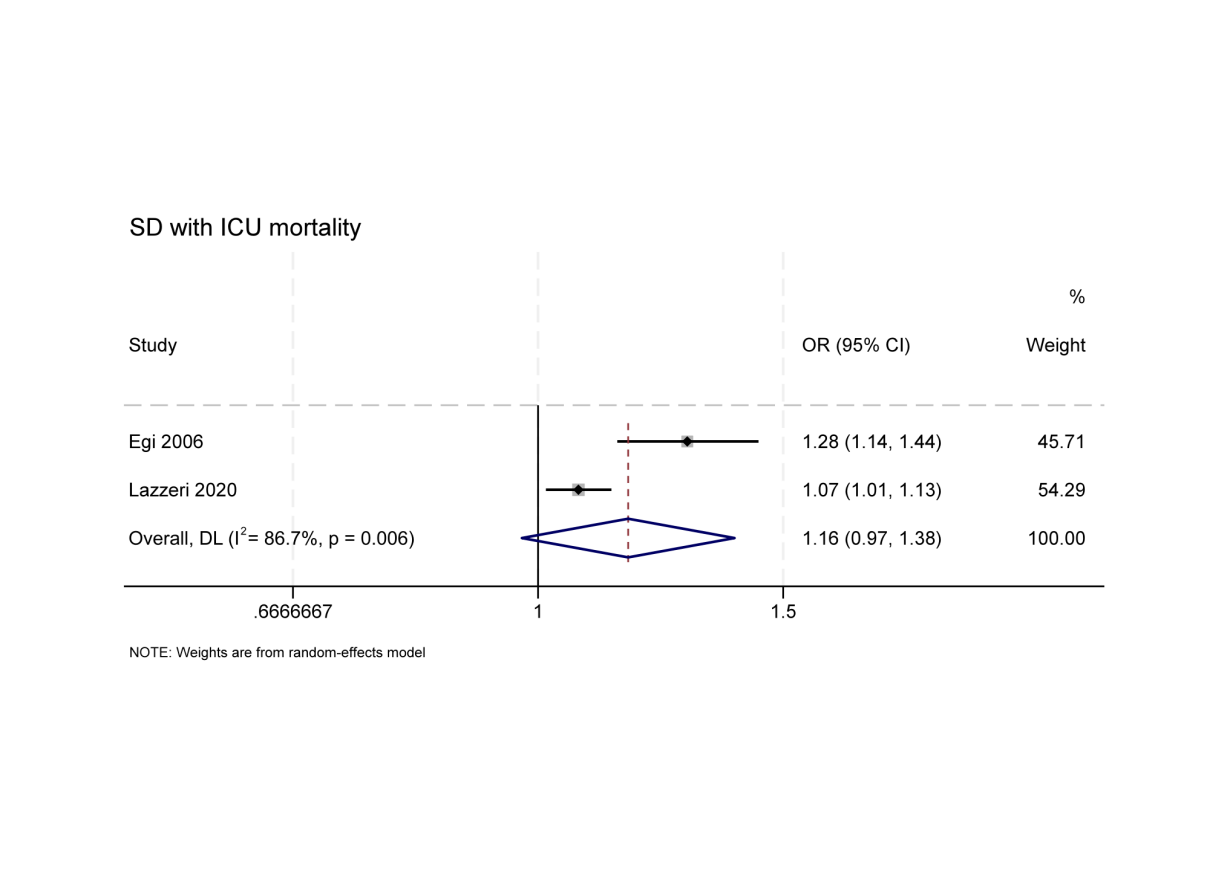


Supplementary Figure 2. Forest plots of SD (per unit increase) and ICU mortality using original OR estimates.


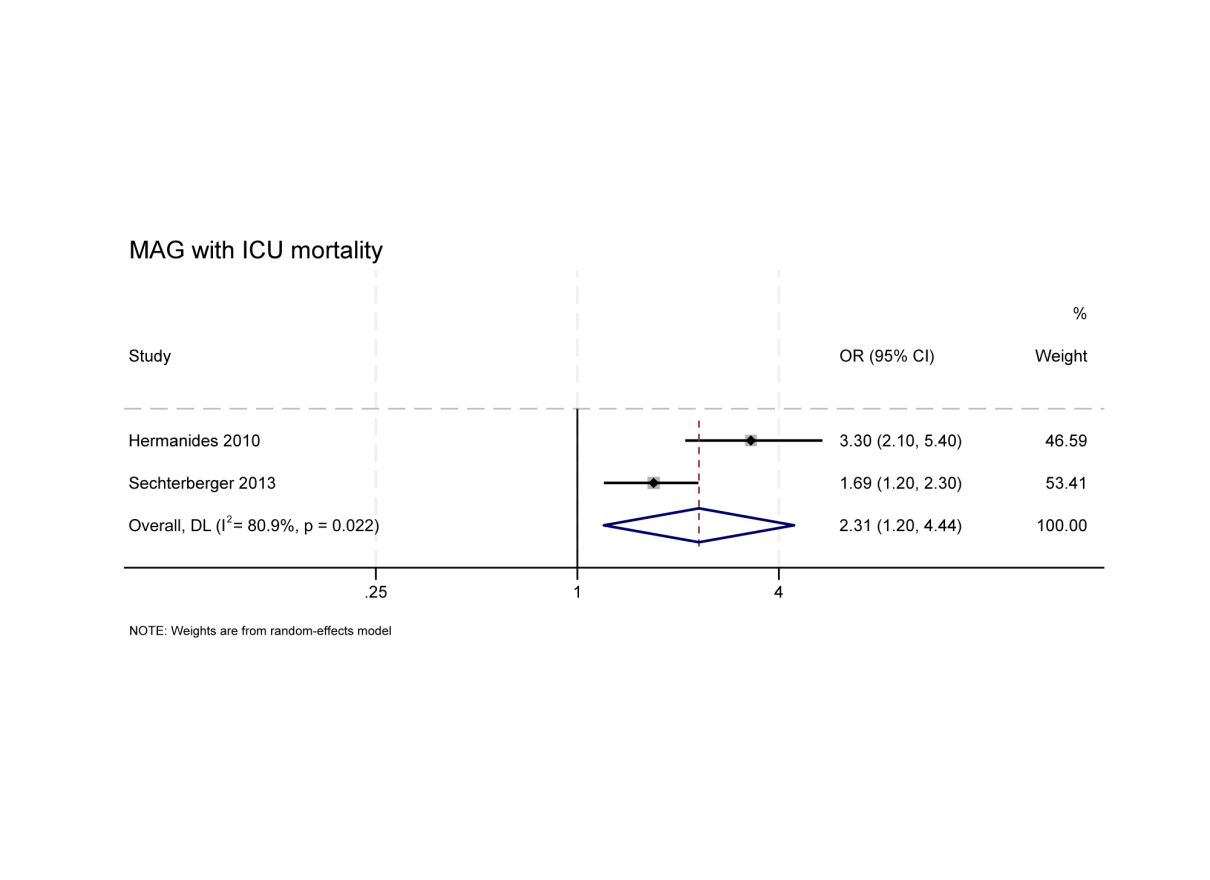


Supplementary Figure 3. Forest plots of MAG (extreme quartiles) and ICU mortality using original OR estimates.


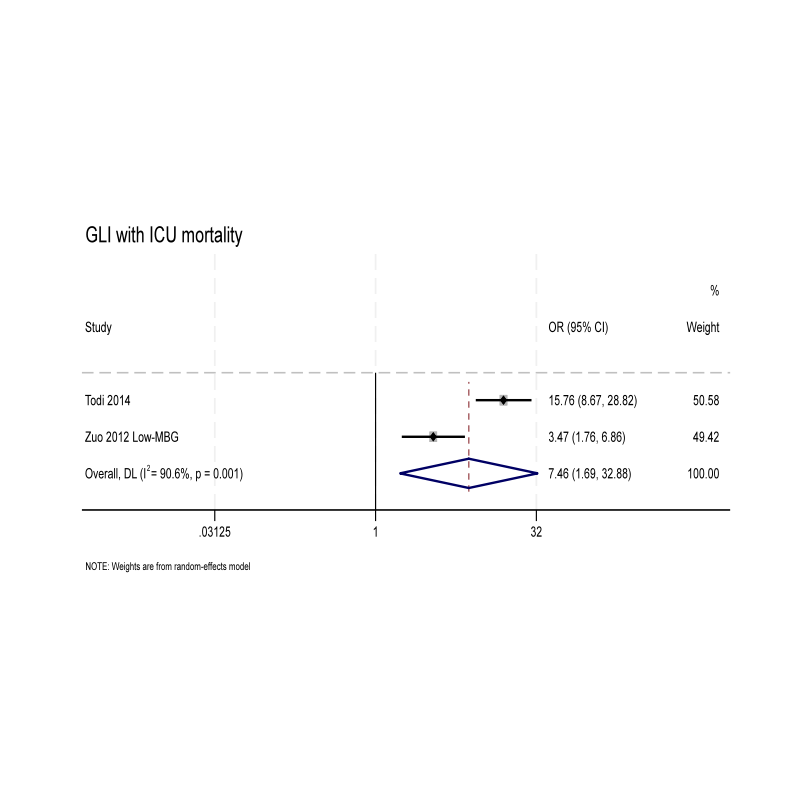


Supplementary Figure 4. Forest plots of GLI (extreme quartiles) and ICU mortality using original OR estimates with harmonized exposure.


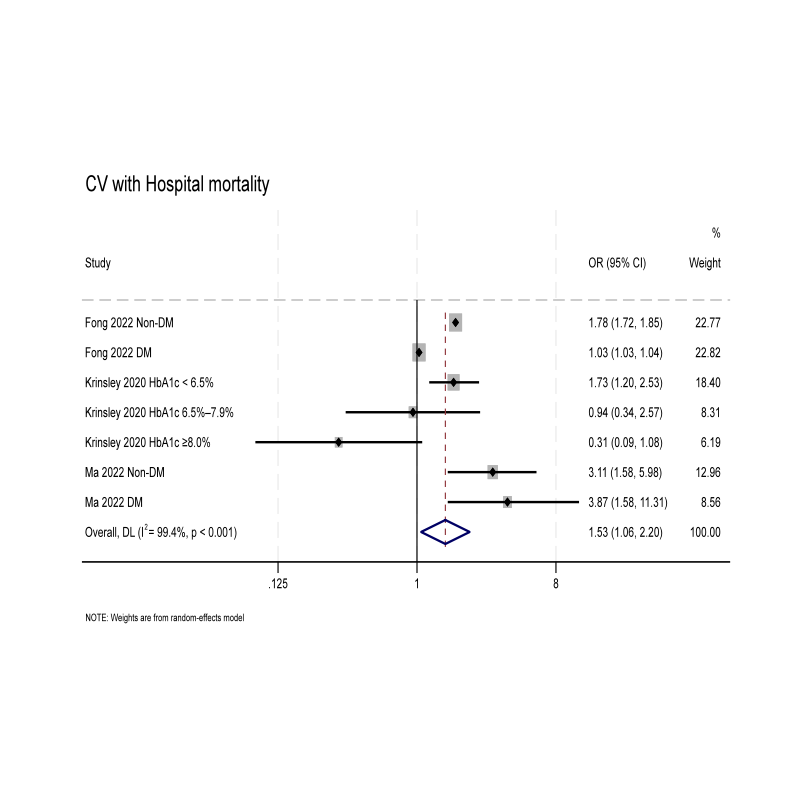


Supplementary Figure 5. Forest plots of CV(extreme quartiles) and hospital mortality using original OR estimates with harmonized exposure.


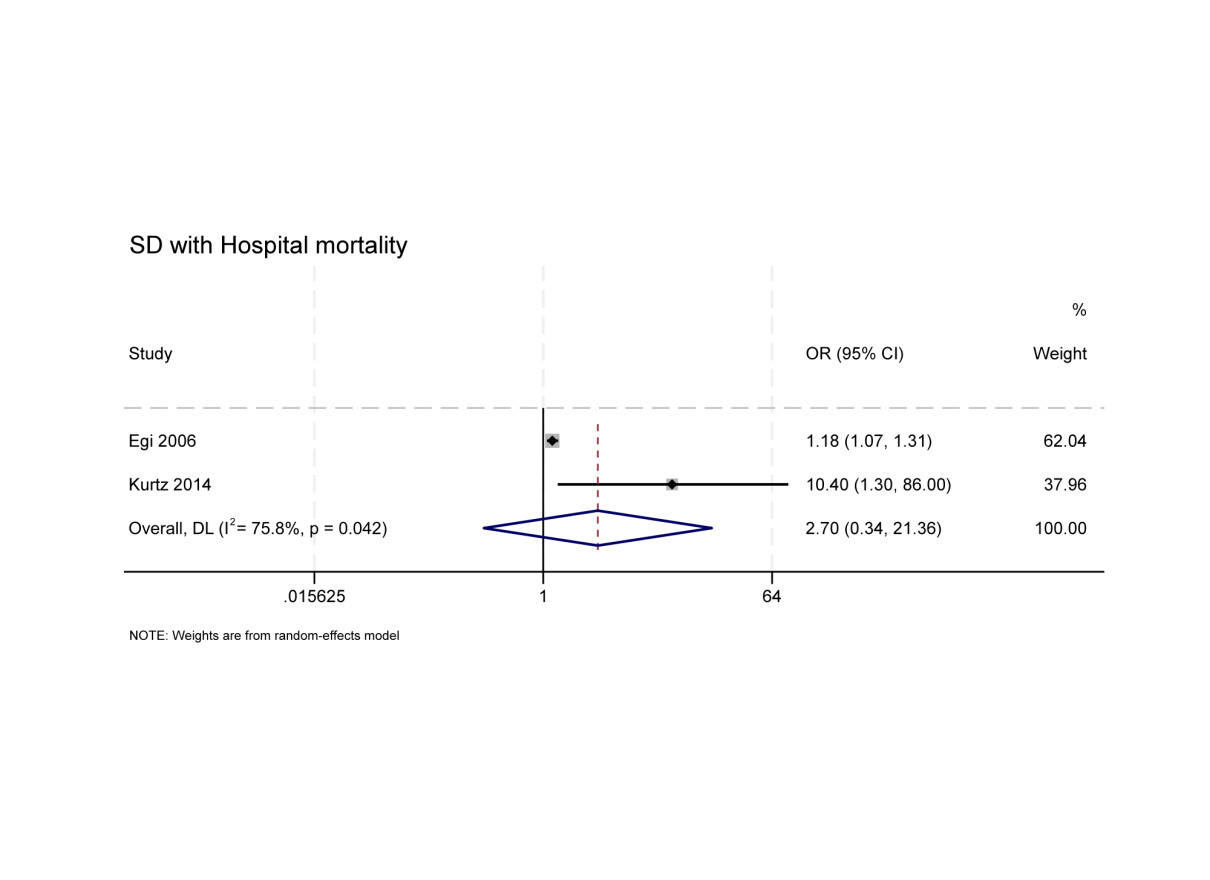


Supplementary Figure 6. Forest plots of SD (per unit increase) and hospital mortality using original OR estimates.
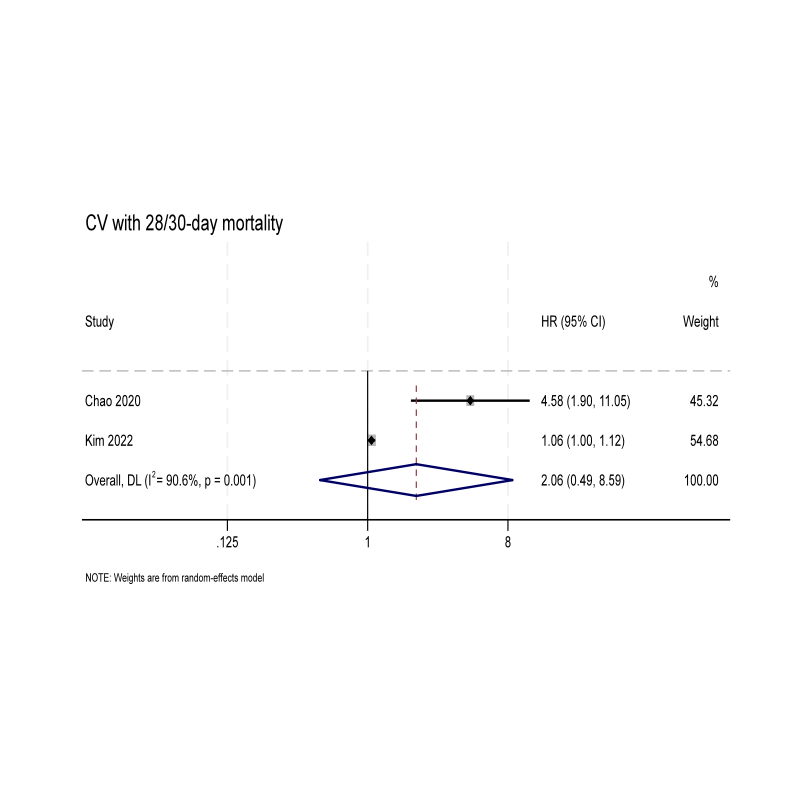


Supplementary Figure 7. Forest plots of CV (extreme quartiles) and 28/30-day mortality using original HR estimates with harmonized exposure.


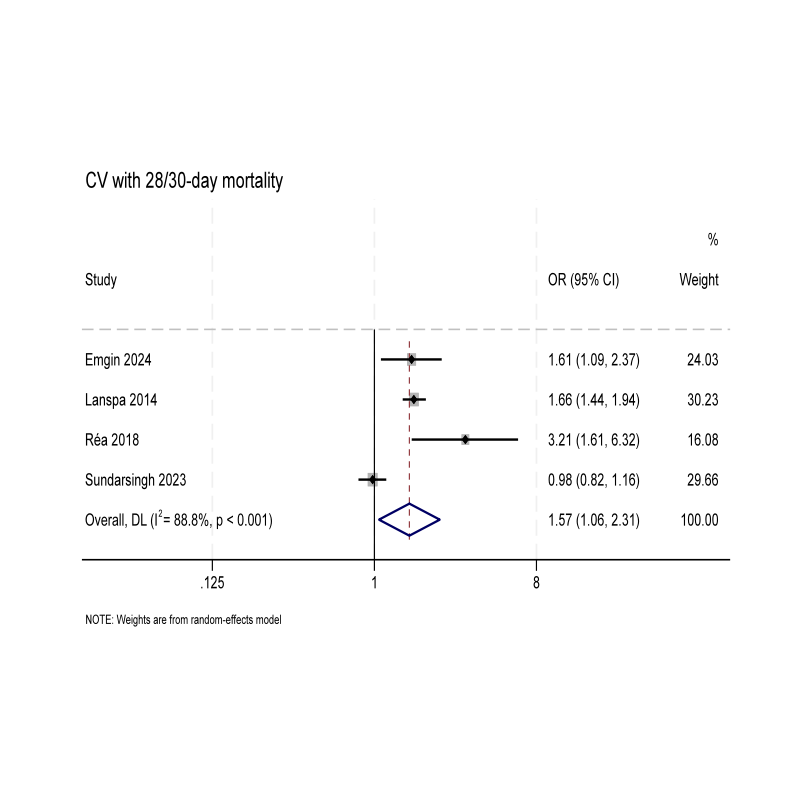


Supplementary Figure 8. Forest plots of CV (extreme quartiles) and 28/30-day mortality using original OR estimates with harmonized exposure.


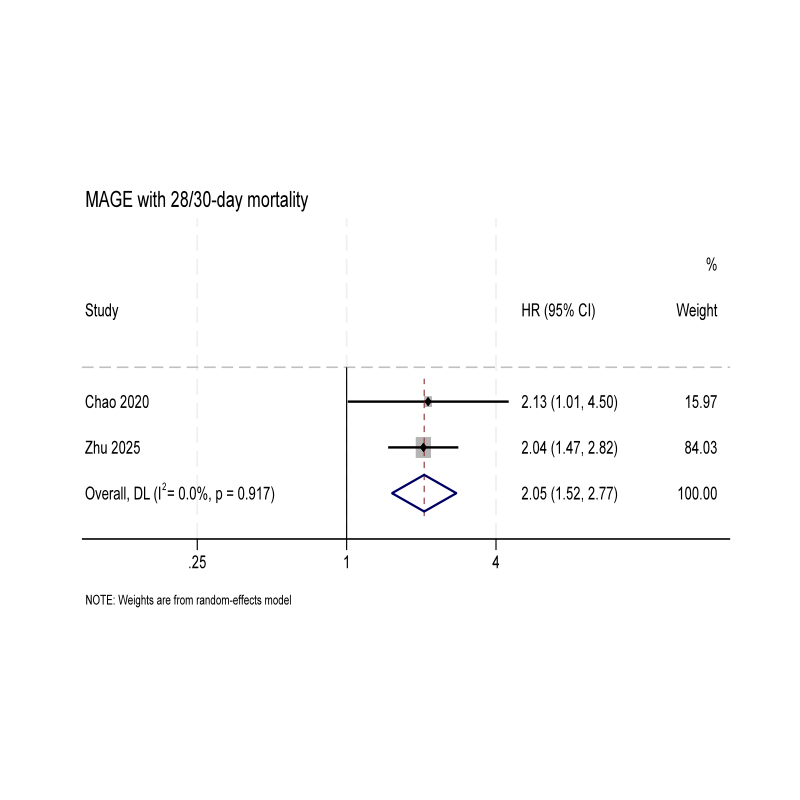


Supplementary Figure 9. Forest plots of MAGE (extreme quartiles) and 28/30-day mortality using original HR estimates with harmonized exposure.


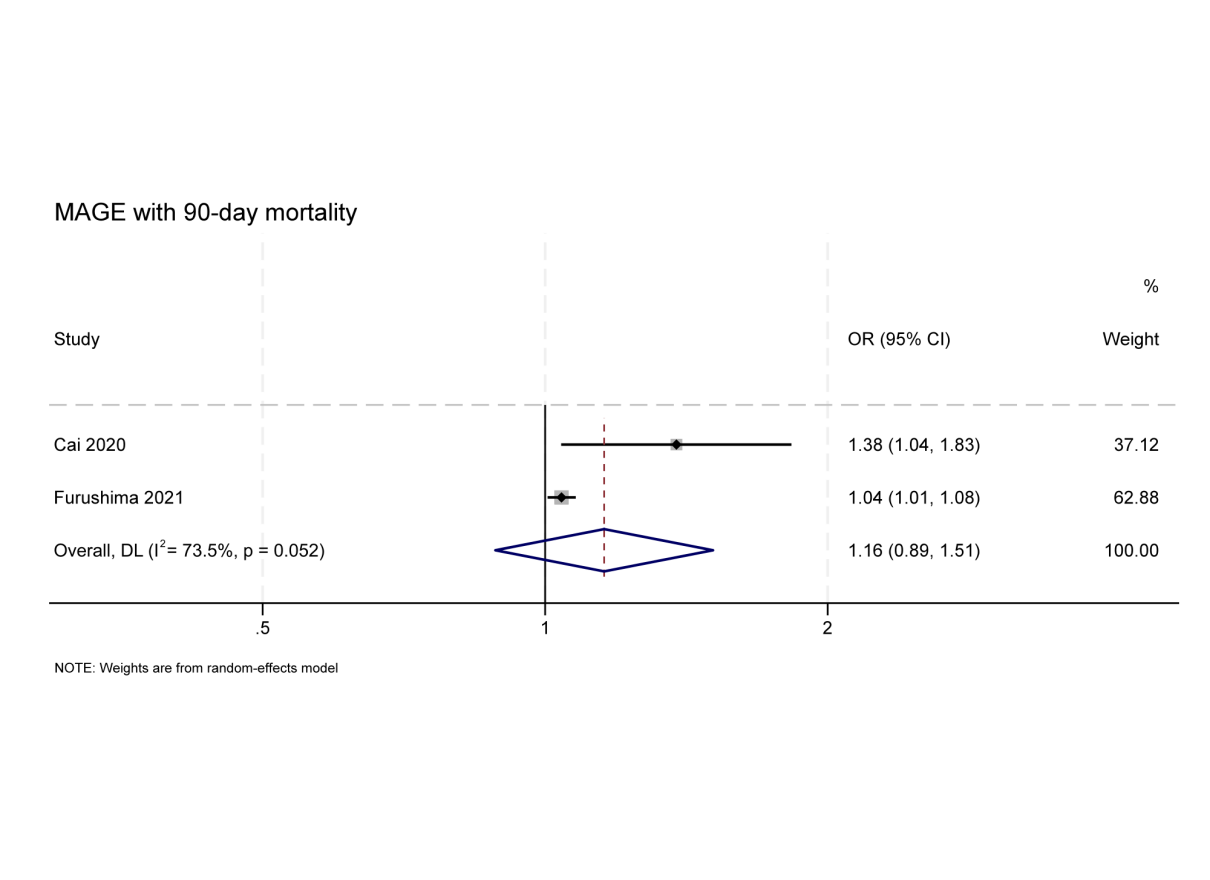


Supplementary Figure 10. Forest plots of MAGE (per unit increase) and 90-day mortality using original OR estimates.


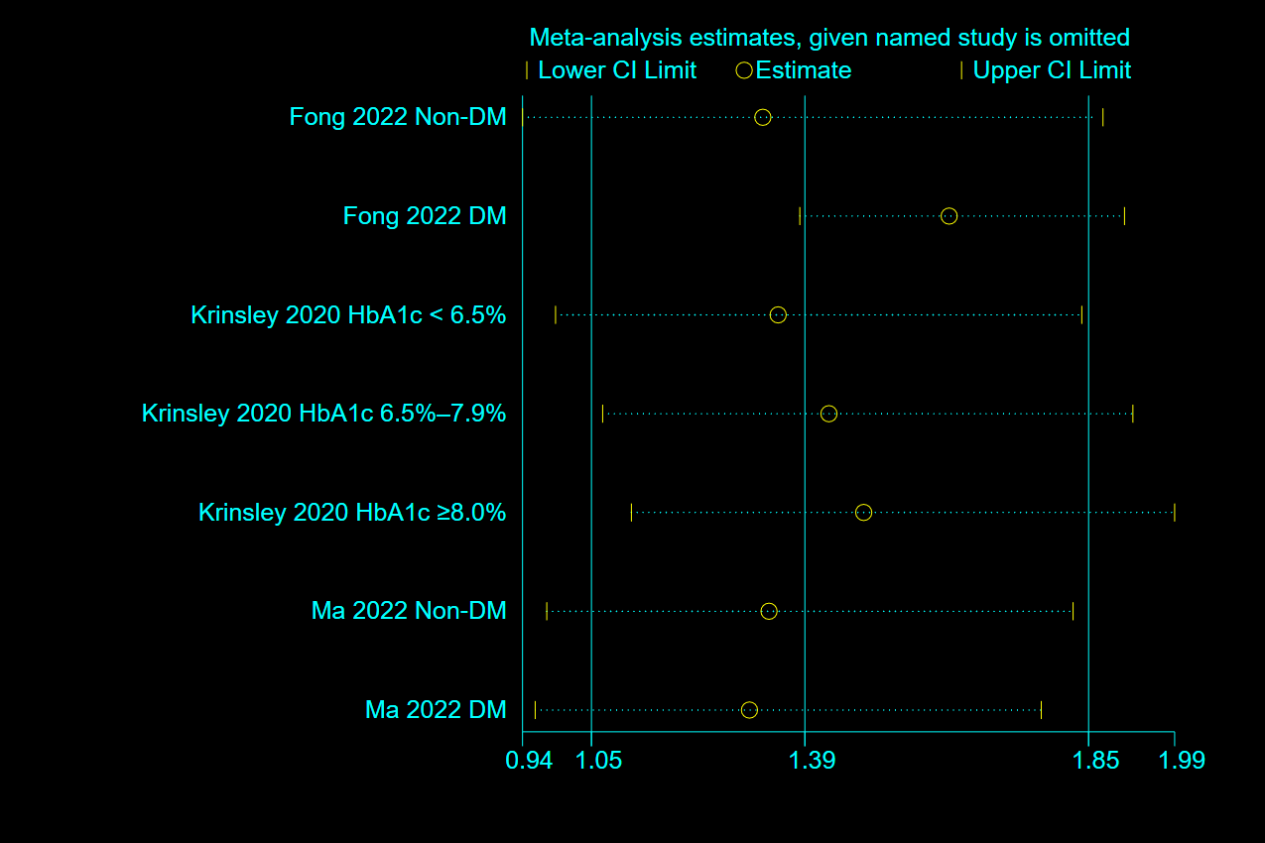


Supplementary Figure 11. Leave-one-out sensitivity analysis of CV for hospital mortality.


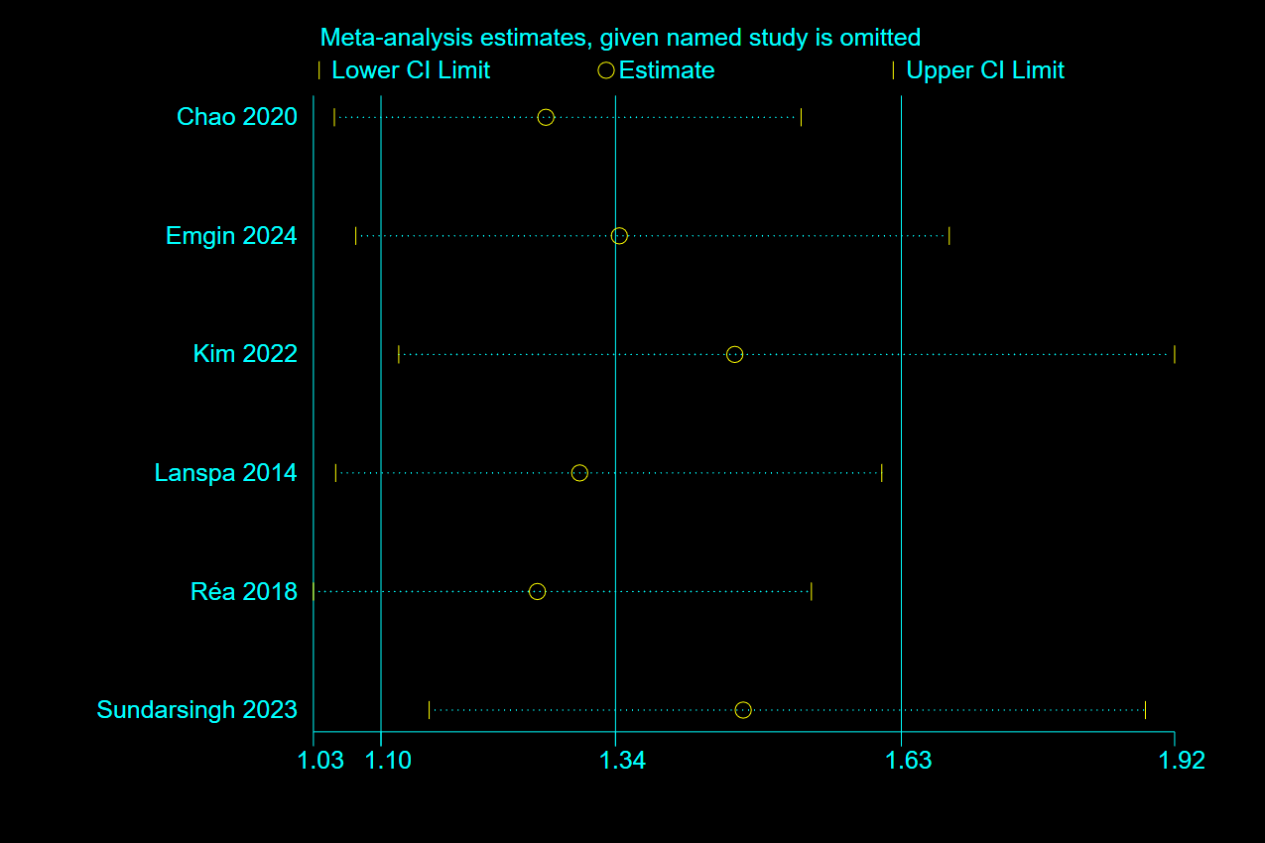


Supplementary Figure 12. Leave-one-out sensitivity analysis of CV for 28/30-day mortality.
